# Supplementary material for: Striatal infusion of cholesterol promotes dose‐dependent behavioral benefits and exerts disease‐modifying effects in Huntington's disease mice
Source: EMBO Mol Med. 2020 Sep 22;12(10):e12519. doi: 10.15252/emmm.202012519 (PMC7539329; doi:10.15252/emmm.202012519)
Supplement: Supplementary file 1 — Appendix [file EMMM-12-e12519-s001.pdf]

Striatal infusion of cholesterol promotes dose-dependent behavioral benefits and exerts disease-modifying effects in Huntington's disease mice

Giulia Birolini[1,2‡], Marta Valenza[1,2‡*], Eleonora Di Paolo[1,2‡], Elena Vezzoli[1,2,†], Francesca Talpo[3], Claudia Maniezzi[3], Claudio Caccia[4], Valerio Leoni[5], Vittoria D. Bocchi[1,2], Paola Conforti[1,2], Elisa Sogne[6], Lara Petricca[7], Cristina Cariulo[7], Margherita Verani[7], Andrea Caricasole[7], Andrea Falqui[6], Gerardo Biella[3], Elena Cattaneo[1,2*]

Appendix – table of content:

Appendix Figure S1
Appendix Figure S2
Appendix Table S3

Appendix Figure S1

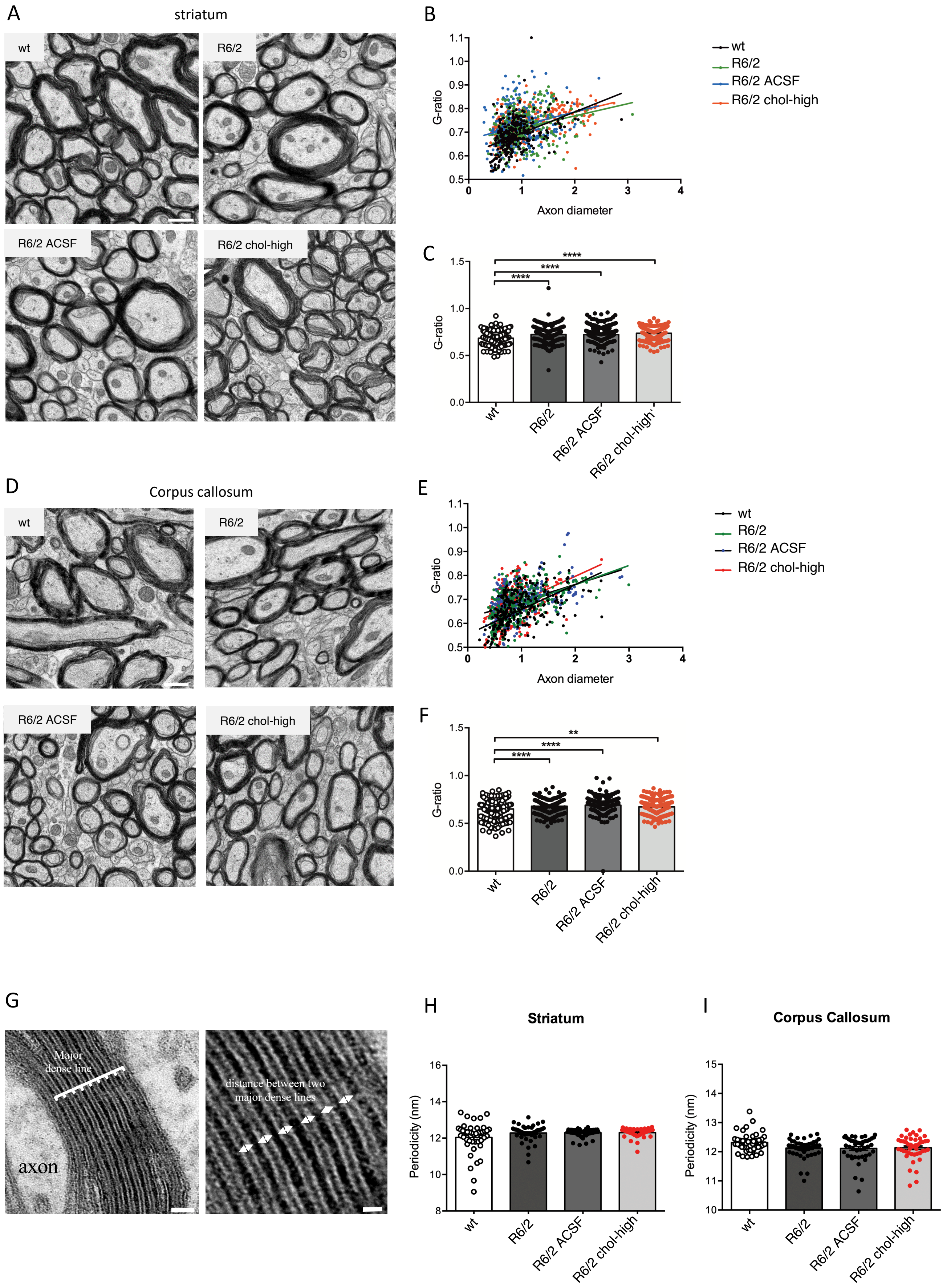

Appendix Figure S2

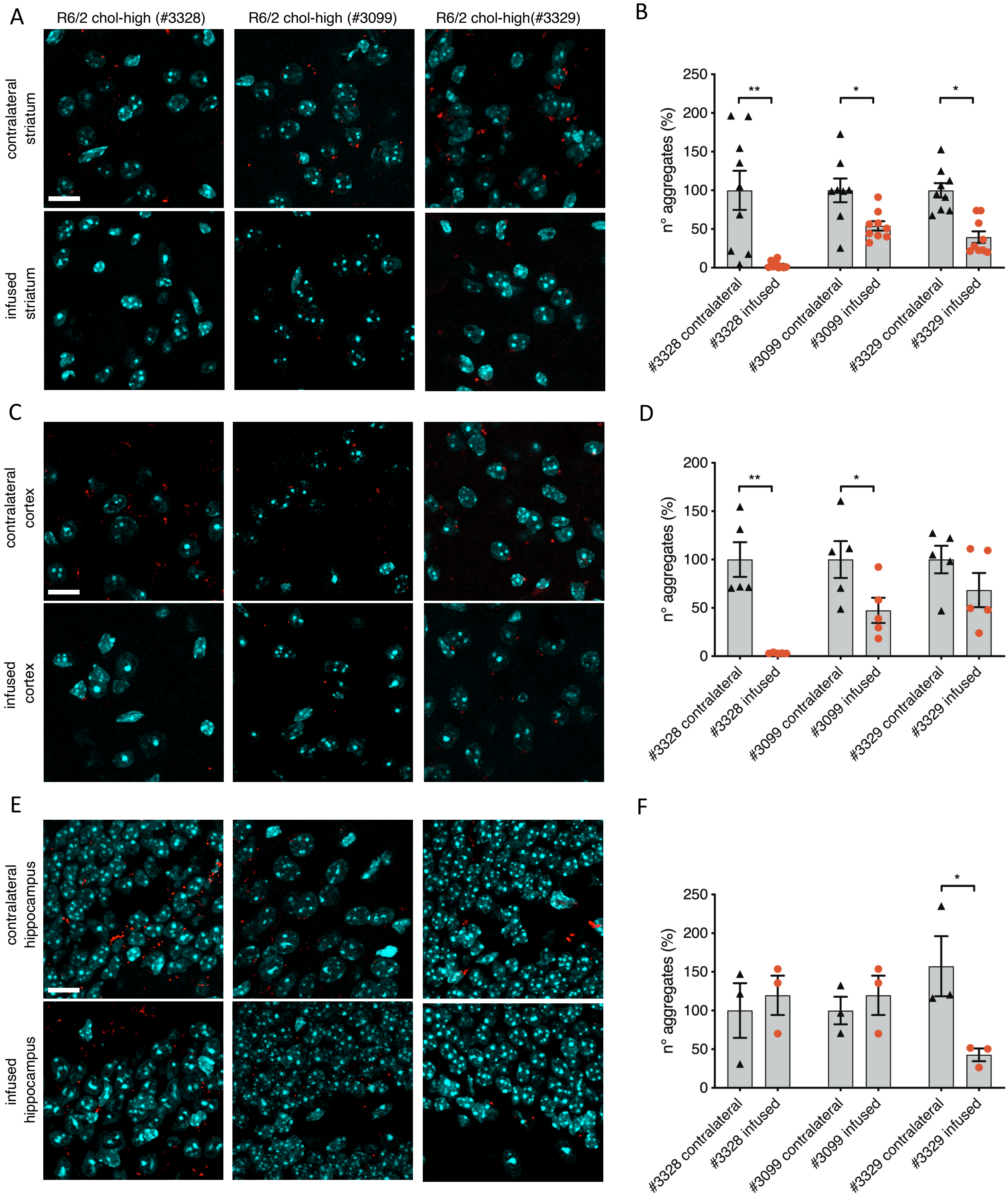

Appendix S3 - Summary of statistics for the main figures

FIGURE 1C			
ANOVA summary			
F	9,635		
P value	< 0,0001		
P value summary	****		
Are differences among means statistically significant? (P < 0.05)	Yes		
R square	0,6769		
Newman-Keuls multiple comparisons test	Mean Diff,	Significant?	Summary
wt vs. R6/2-ACSF	15,05	Yes	**
wt vs. R6/2-chol (low)	6,229	No	ns
wt vs. R6/2-chol (medium)	-6,886	No	ns
wt vs. R6/2-chol (high)	-9,614	No	ns
R6/2-ACSF vs. R6/2-chol (low)	-8,819	No	ns
R6/2-ACSF vs. R6/2-chol (medium)	-21,93	Yes	**
R6/2-ACSF vs. R6/2-chol (high)	-24,66	Yes	***
R6/2-chol (low) vs. R6/2-chol (medium)	-13,12	No	ns
R6/2-chol (low) vs. R6/2-chol (high)	-15,84	Yes	*
R6/2-chol (medium) vs. R6/2-chol (high)	-2,727	No	ns

FIGURE 1D			
ANOVA summary - Rotarod 8 weeks			
F	5,132		
P value	0,001		
P value summary	***		
Are differences among means statistically significant? (P < 0.05)	Yes		
R square	0,2002		
Newman-Keuls multiple comparisons test	Mean Diff,	Significant?	Summary
wt vs. R6/2-ACSF	87,77	Yes	***
wt vs. R6/2-chol (low)	80,01	Yes	**
wt vs. R6/2-chol (medium)	78,59	Yes	*
wt vs. R6/2-chol (high)	51,56	Yes	*
R6/2-ACSF vs. R6/2-chol (low)	-7,767	No	ns
R6/2-ACSF vs. R6/2-chol (medium)	-9,188	No	ns
R6/2-ACSF vs. R6/2-chol (high)	-36,21	No	ns
R6/2-chol (low) vs. R6/2-chol (medium)	-1,421	No	ns
R6/2-chol (low) vs. R6/2-chol (high)	-28,45	No	ns
R6/2-chol (medium) vs. R6/2-chol (high)	-27,02	No	ns
ANOVA summary - Rotarod 10 weeks			
F	33,78		
P value	< 0,0001		
P value summary	****		
Are differences among means statistically significant? (P < 0.05)	Yes		
R square	0,634		
Newman-Keuls multiple comparisons test	Mean Diff,	Significant?	Summary
wt vs. R6/2-ACSF	161,4	Yes	****
wt vs. R6/2-chol (low)	170,6	Yes	****
wt vs. R6/2-chol (medium)	158,1	Yes	****
wt vs. R6/2-chol (high)	116,6	Yes	****
R6/2-ACSF vs. R6/2-chol (low)	9,271	No	ns
R6/2-ACSF vs. R6/2-chol (medium)	-3,283	No	ns
R6/2-ACSF vs. R6/2-chol (high)	-44,72	Yes	*
R6/2-chol (low) vs. R6/2-chol (medium)	-12,55	No	ns
R6/2-chol (low) vs. R6/2-chol (high)	-53,99	Yes	*
R6/2-chol (medium) vs. R6/2-chol (high)	-41,44	No	ns

FIGURE 1E			
ANOVA summary - Global activity 11 weeks			
F	13,45		
P value	<0,0001		
P value summary	****		

Significant diff. among means (P < 0.05)?	Yes		
R squared	0,4476		
Newman-Keuls multiple comparisons test	Mean Diff,	Significant?	Summary
wt vs. R6/2-ASCF	5571	Yes	****
wt vs. R6/2-chol (low)	6087	Yes	****
wt vs. R6/2-chol (medium)	4561	Yes	**
wt vs. R6/2-chol (high)	1555	No	ns
R6/2-ASCF vs. R6/2-chol (low)	515,8	No	ns
R6/2-ASCF vs. R6/2-chol (medium)	-1010	No	ns
R6/2-ASCF vs. R6/2-chol (high)	-4016	Yes	***
R6/2-chol (low) vs. R6/2-chol (medium)	-1526	No	ns
R6/2-chol (low) vs. R6/2-chol (high)	-4532	Yes	***
R6/2-chol (medium) vs. R6/2-chol (high)	-3006	Yes	*

**FIGURE 1F**			
ANOVA summary - Distance 11 weeks			
F	13,17		
P value	<0,0001		
P value summary	****		
Significant diff. among means (P < 0.05)?	Yes		
R squared	0,4126		
Newman-Keuls multiple comparisons test	Mean Diff,	Significant?	Summary
wt vs. R6/2-ASCF	7035	Yes	****
wt vs. R6/2-chol (low)	7478	Yes	****
wt vs. R6/2-chol (medium)	6050	Yes	**
wt vs. R6/2-chol (high)	3380	Yes	**
R6/2-ASCF vs. R6/2-chol (low)	442,2	No	ns
R6/2-ASCF vs. R6/2-chol (medium)	-985,1	No	ns
R6/2-ASCF vs. R6/2-chol (high)	-3656	Yes	**
R6/2-chol (low) vs. R6/2-chol (medium)	-1427	No	ns
R6/2-chol (low) vs. R6/2-chol (high)	-4098	Yes	*
R6/2-chol (medium) vs. R6/2-chol (high)	-2671	No	ns

**FIGURE 1G**			
ANOVA summary - Vel Mean 11 weeks			
F	16,58		
P value	<0,0001		
P value summary	****		
Significant diff. among means (P < 0.05)?	Yes		
R squared	0,4693		
Newman-Keuls multiple comparisons test	Mean Diff,	Significant?	Summary
wt vs. R6/2-ASCF	2,042	Yes	****
wt vs. R6/2-chol (low)	2,238	Yes	****
wt vs. R6/2-chol (medium)	1,283	Yes	**
wt vs. R6/2-chol (high)	1,042	Yes	**
R6/2-ASCF vs. R6/2-chol (low)	0,1964	No	ns
R6/2-ASCF vs. R6/2-chol (medium)	-0,7584	No	ns
R6/2-ASCF vs. R6/2-chol (high)	-0,9995	Yes	**
R6/2-chol (low) vs. R6/2-chol (medium)	-0,9548	No	ns
R6/2-chol (low) vs. R6/2-chol (high)	-1,196	Yes	**
R6/2-chol (medium) vs. R6/2-chol (high)	-0,2411	No	ns

**FIGURE 1H**			
ANOVA summary - Stereotyped Mov 11 weeks			
F	14,63		
P value	<0,0001		
P value summary	****		
Significant diff. among means (P < 0.05)?	Yes		
R squared	0,4383		
Newman-Keuls multiple comparisons test	Mean Diff,	Significant?	Summary
wt vs. R6/2-ASCF	442,1	Yes	****
wt vs. R6/2-chol (low)	555,2	Yes	****
wt vs. R6/2-chol (medium)	421	Yes	***
wt vs. R6/2-chol (high)	206,3	Yes	*
R6/2-ASCF vs. R6/2-chol (low)	113,1	No	ns
R6/2-ASCF vs. R6/2-chol (medium)	-21,08	No	ns
R6/2-ASCF vs. R6/2-chol (high)	-235,8	Yes	*

R6/2-chol (low) vs. R6/2-chol (medium)	-134,2	No	ns
R6/2-chol (low) vs. R6/2-chol (high)	-348,9	Yes	**
R6/2-chol (medium) vs. R6/2-chol (high)	-214,8	No	ns

**FIGURE 1I**			
ANOVA summary - Rearings 11 weeks			
F	16,59		
P value	<0,0001		
P value summary	****		
Significant diff. among means (P < 0.05)?	Yes		
R squared	0,4694		
Newman-Keuls multiple comparisons test	Mean Diff,	Significant?	Summary
wt vs. R6/2-ASCF	407,7	Yes	****
wt vs. R6/2-chol (low)	378,3	Yes	****
wt vs. R6/2-chol (medium)	350,5	Yes	***
wt vs. R6/2-chol (high)	288,3	Yes	****
R6/2-ASCF vs. R6/2-chol (low)	-29,42	No	ns
R6/2-ASCF vs. R6/2-chol (medium)	-57,23	No	ns
R6/2-ASCF vs. R6/2-chol (high)	-119,5	No	ns
R6/2-chol (low) vs. R6/2-chol (medium)	-27,81	No	ns
R6/2-chol (low) vs. R6/2-chol (high)	-90,04	No	ns
R6/2-chol (medium) vs. R6/2-chol (high)	-62,23	No	ns
R6/2-chol (high) vs R6/2-ACSF			
Unpaired t test			
P value	0,0419		
P value summary	#		
Significantly different (P < 0.05)?	Yes		
One- or two-tailed P value?	Two-tailed		
t, df	t=2,108, df=37		

**FIGURE 1K**			
ANOVA summary - Center vs Periphery 11 weeks			
F	10,06		
P value	<0,0001		
P value summary	****		
Significant diff. among means (P < 0.05)?	Yes		
R squared	0,3651		
Newman-Keuls multiple comparisons test	Mean Diff,	Significant?	Summary
wt vs. R6/2-ACSF	12,25	Yes	***
wt vs. R6/2-chol (low)	14,96	Yes	**
wt vs. R6/2-chol (medium)	8,864	No	ns
wt vs. R6/2-chol (high)	-5,886	No	ns
R6/2-ACSF vs. R6/2-chol (low)	2,717	No	ns
R6/2-ACSF vs. R6/2-chol (medium)	-3,383	No	ns
R6/2-ACSF vs. R6/2-chol (high)	-18,13	Yes	****
R6/2-chol (low) vs. R6/2-chol (medium)	-6,1	No	ns
R6/2-chol (low) vs. R6/2-chol (high)	-20,85	Yes	***
R6/2-chol (medium) vs. R6/2-chol (high)	-14,75	Yes	**

**FIGURE 1L**			
ANOVA summary - NORT 11 weeks			
F	6,031		
P value	0,0003		
P value summary	***		
Significant diff. among means (P < 0.05)?	Yes		
R squared	0,2434		
Newman-Keuls multiple comparisons test	Mean Diff,	Significant?	Summary
wt vs. R6/2-ACSF	31,91	Yes	***
wt vs. R6/2-chol (low)	3,245	No	ns
wt vs. R6/2-chol (medium)	3,101	No	ns
wt vs. R6/2-chol (high)	2,28	No	ns
R6/2-ACSF vs. R6/2-chol (low)	-28,66	Yes	**
R6/2-ACSF vs. R6/2-chol (medium)	-28,81	Yes	*
R6/2-ACSF vs. R6/2-chol (high)	-29,63	Yes	**
R6/2-chol (low) vs. R6/2-chol (medium)	-0,1444	No	ns
R6/2-chol (low) vs. R6/2-chol (high)	-0,9654	No	ns
R6/2-chol (medium) vs. R6/2-chol (high)	-0,8209	No	ns

FIGURE 2D			
ANOVA summary			
F	37,74		
P value	<0,0001		
P value summary	****		
Significant diff. among means (P < 0.05)?	Yes		
R squared	0,8531		
Newman-Keuls multiple comparisons test	Mean Diff,	Significant?	Summary
wt vs. R6/2-ACSF	1,817	Yes	****
wt vs. R6/2-chol	1,189	Yes	****
R6/2-ACSF vs. R6/2-chol	-0,6276	Yes	*

FIGURE 2G			
ANOVA summary			
F	10,15		
P value	<0,0001		
P value summary	****		
Significant diff. among means (P < 0.05)?	Yes		
R squared	0,14		
Newman-Keuls multiple comparisons test	Mean Diff,	Significant?	Summary
wt vs. R6/2	0,1492	Yes	***
wt vs. R6/2-ACSF	0,1537	Yes	***
wt vs. R6/2-chol	0,2027	Yes	****
R6/2 vs. R6/2-ACSF	0,004505	No	ns
R6/2 vs. R6/2-chol	0,05353	No	ns
R6/2-ACSF vs. R6/2-chol	0,04903	No	ns

FIGURE 2J			
ANOVA summary			
F	20,24		
P value	<0,0001		
P value summary	****		
Significant diff. among means (P < 0.05)?	Yes		
R squared	0,2089		
Newman-Keuls multiple comparisons test	Mean Diff,	Significant?	Summary
wt vs. R6/2	42	Yes	****
wt vs. R6/2 ACSF	33,38	Yes	****
wt vs. R6/2-chol	12,77	Yes	*
R6/2 vs. R6/2 ACSF	-8,618	No	ns
R6/2 vs. R6/2-chol	-29,23	Yes	****
R6/2 ACSF vs. R6/2-chol	-20,61	Yes	***

FIGURE 2K			
ANOVA summary			
F	5,643		
P value	0,0009		
P value summary	***		
Significant diff. among means (P < 0.05)?	Yes		
R squared	0,06693		
Newman-Keuls multiple comparisons test	Mean Diff,	Significant?	Summary
wt vs. R6/2	1,203	Yes	*
wt vs. R6/2-ACSF	1,154	Yes	**
wt vs. R6/2-chol	0,1562	No	ns
R6/2 vs. R6/2-ACSF	-0,04863	No	ns
R6/2 vs. R6/2-chol	-1,046	Yes	*
R6/2-ACSF vs. R6/2-chol	-0,9978	Yes	**

FIGURE 3C			
ANOVA summary			
F	14,85		
P value	0,0003		
P value summary	***		
Significant diff. among means (P < 0.05)?	Yes		
R squared	0,6644		
Newman-Keuls multiple comparisons test	Mean Diff,	Significant?	Summary

wt vs. R6/2-ACSF	-1,212	Yes	***
wt vs. R6/2-chol	0,06288	No	ns
R6/2-ACSF vs. R6/2-chol	1,275	Yes	***

**FIGURE 3E**			
ANOVA summary			
F	8,363		
P value	0,0001		
P value summary	***		
Significant diff. among means (P < 0.05)?	Yes		
R squared	0,3213		
Newman-Keuls multiple comparisons test	Mean Diff,	Significant?	Summary
wt vs. R6/2	-0,01566	Yes	***
wt vs. R6/2-ACSF	-0,013	Yes	**
wt vs. R6/2-chol	-0,002188	No	ns
R6/2 vs. R6/2-ACSF	0,002663	No	ns
R6/2 vs. R6/2-chol	0,01348	Yes	**
R6/2-ACSF vs. R6/2-chol	0,01081	Yes	**

**FIGURE 4B**			
ANOVA summary - contralateral stiatum			
F	67,45		
P value	< 0,0001		
P value summary	****		
Are differences among means statistically significant? (P < 0.05)	Yes		
R square	0,9375		
Newman-Keuls multiple comparisons test	Mean Diff,	Significant?	Summary
wt vs. R6/2-ASCF	29,55	Yes	****
wt vs. R6/2-Chol	44,2	Yes	****
R6/2-ASCF vs. R6/2-Chol	14,64	Yes	**
ANOVA summary - infused striatum			
F	23,75		
P value	0,0003		
P value summary	***		
Are differences among means statistically significant? (P < 0.05)	Yes		
R square	0,8407		
Newman-Keuls multiple comparisons test	Mean Diff,	Significant?	Summary
wt vs. R6/2-ASCF	19,74	Yes	**
wt vs. R6/2-Chol	-18,3	Yes	**
R6/2-ASCF vs. R6/2-Chol	-38,04	Yes	***

**FIGURE 4C**			
ANOVA summary - contralateral stiatum			
F	89,53		
P value	< 0,0001		
P value summary	****		
Are differences among means statistically significant? (P < 0.05)	Yes		
R square	0,9521		
Newman-Keuls multiple comparisons test	Mean Diff,	Significant?	Summary
wt vs. R6/2-ASCF	76,57	Yes	****
wt vs. R6/2-Chol	76,88	Yes	****
R6/2-ASCF vs. R6/2-Chol	0,3097	No	ns
ANOVA summary - infused striatum			
F	52,46		
P value	< 0,0001		
P value summary	****		
Are differences among means statistically significant? (P < 0.05)	Yes		
R square	0,921		
Newman-Keuls multiple comparisons test	Mean Diff,	Significant?	Summary
wt vs. R6/2-ASCF	73,2	Yes	****

wt vs. R6/2-Chol	46,2	Yes	***
R6/2-ASCF vs. R6/2-Chol	-27	Yes	**

**FIGURE 4D**			
ANOVA summary - contralateral stiatum			
F	212,1		
P value	< 0,0001		
P value summary	****		
Are differences among means statistically significant? (P < 0.05)	Yes		
R square	0,9792		
Newman-Keuls multiple comparisons test	Mean Diff,	Significant?	Summary
wt vs. R6/2-ASCF	71,21	Yes	****
wt vs. R6/2-Chol	69,49	Yes	****
R6/2-ASCF vs. R6/2-Chol	-1,711	No	ns
ANOVA summary - infused striatum			
F	23,16		
P value	0,0003		
P value summary	***		
Are differences among means statistically significant? (P < 0.05)	Yes		
R square	0,8373		
Newman-Keuls multiple comparisons test	Mean Diff,	Significant?	Summary
wt vs. R6/2-ASCF	59,22	Yes	***
wt vs. R6/2-Chol	8,048	No	ns
R6/2-ASCF vs. R6/2-Chol	-51,17	Yes	***

**FIGURE 4E**			
ANOVA summary - contralateral stiatum			
F	8,488		
P value	0,0085		
P value summary	**		
Are differences among means statistically significant? (P < 0.05)	Yes		
R square	0,6535		
Newman-Keuls multiple comparisons test	Mean Diff,	Significant?	Summary
wt vs. R6/2-ASCF	26,6	Yes	*
wt vs. R6/2-Chol	32,73	Yes	**
R6/2-ASCF vs. R6/2-Chol	6,137	No	ns
ANOVA summary - infused striatum			
F	9,097		
P value	0,0069		
P value summary	**		
Are differences among means statistically significant? (P < 0.05)	Yes		
R square	0,669		
Newman-Keuls multiple comparisons test	Mean Diff,	Significant?	Summary
wt vs. R6/2-ASCF	11,66	No	ns
wt vs. R6/2-Chol	-35,89	Yes	*
R6/2-ASCF vs. R6/2-Chol	-47,55	Yes	**

**FIGURE 4G and 4I**			
R6/2 vs wt			
Unpaired t test			
P value	<0,0001		
P value summary	****		
Significantly different (P < 0.05)?	Yes		
One- or two-tailed P value?	Two-tailed		
t, df	t=8,618, df=8		
R6/2 chol high (infused) vs R6/2 chol high (contralateral)			
Unpaired t test			
P value	0,001		
P value summary	**		
Significantly different (P < 0.05)?	Yes		

One- or two-tailed P value?	Two-tailed		
t, df	t=4,432, df=11		

**FIGURE 5B**			
ANOVA summary			
F	12,14		
P value	<0,0001		
P value summary	****		
Significant diff. among means (P < 0.05)?	Yes		
R squared	0,2651		
Newman-Keuls multiple comparisons test	Mean Diff,	Significant?	Summary
ACSF contralateral vs. ACSF infused	14,74	No	ns
ACSF contralateral vs. Chol contralateral	-4,615E-10	No	ns
ACSF contralateral vs. Chol infused	67,62	Yes	****
ACSF infused vs. Chol contralateral	-14,74	No	ns
ACSF infused vs. Chol infused	52,88	Yes	****
Chol contralateral vs. Chol infused	67,62	Yes	****

**FIGURE 5C**			
ANOVA summary			
F	4,5		
P value	0,0053		
P value summary	**		
Significant diff. among means (P < 0.05)?	Yes		
R squared	0,1179		
Newman-Keuls multiple comparisons test	Mean Diff,	Significant?	Summary
ACSF contralateral vs. ACSF infused	-0,2515	No	ns
ACSF contralateral vs. Chol contralateral	-0,27	No	ns
ACSF contralateral vs. Chol infused	0,9782	Yes	*
ACSF infused vs. Chol contralateral	-0,01846	No	ns
ACSF infused vs. Chol infused	1,23	Yes	**
Chol contralateral vs. Chol infused	1,248	Yes	*

**FIGURE 5E**			
Chol infused vs Chol contralateral			
Unpaired t test			
P value	<0,0001		
P value summary	****		
Significantly different (P < 0.05)?	Yes		
One- or two-tailed P value?	Two-tailed		
t, df	t=5,281, df=28		

**FIGURE 5G**			
Chol infused vs Chol contralateral			
Unpaired t test			
P value	<0,0001		
P value summary	****		
Significantly different (P < 0.05)?	Yes		
One- or two-tailed P value?	Two-tailed		
t, df	t=4,738, df=28		

**FIGURE 5L**			
R6/2-Chol vs R6/2-ACSF			
Unpaired t test with Welch's correction			
P value	0,1594		
P value summary	ns		
Significantly different? (P < 0.05)	No		
One- or two-tailed P value?	Two-tailed		
Welch-corrected t, df	t=1,689 df=4,430		
How big is the difference?			
Mean ± SEM of column A	0,01035 ± 0,001016, n=5		
Mean ± SEM of column B	0,00859 ± 0,0002371, n=4		
Difference between means	-0,001762 ± 0,001043		
95% confidence interval	-0,004550 to 0,001026		
R squared	0,3918		
F test to compare variances			
F,DFn, Dfd	22,95, 4, 3		

P value	0,0276		
P value summary	*		
Significantly different? (P < 0.05)	Yes		

**FIGURE 5M**			
R6/2-Chol vs R6/2-ACSF			
Unpaired t test			
P value	0,0481		
P value summary	*		
Significantly different? (P < 0.05)	Yes		
One- or two-tailed P value?	Two-tailed		
t, df	t=2,390 df=7		
How big is the difference?			
Mean ± SEM of column A	0,01716 ± 0,002016, n=5		
Mean ± SEM of column B	0,01083 ± 0,001526, n=4		
Difference between means	-0,006334 ± 0,002650		
95% confidence interval	-0,01260 to -6,843e-005		
R squared	0,4494		
F test to compare variances			
F,DFn, Dfd	2,183, 4, 3		
P value	0,5473		
P value summary	ns		
Significantly different? (P < 0.05)	No		

**FIGURE 6B**			
ANOVA summary			
F	22,35		
P value	<0,0001		
P value summary	****		
Significant diff. among means (P < 0.05)?	Yes		
R squared	0,5828		
Newman-Keuls multiple comparisons test	Mean Diff,	Significant?	Summary
wt vs. ACSF contralateral	-92,4	Yes	****
wt vs. ACSF infused	-98,63	Yes	****
wt vs. Chol contralateral	-92,4	Yes	****
wt vs. Chol infused	-104,3	Yes	****
ACSF contralateral vs. ACSF infused	-6,231	No	ns
ACSF contralateral vs. Chol contralateral	-0,00001	No	ns
ACSF contralateral vs. Chol infused	-11,86	No	ns
ACSF infused vs. Chol contralateral	6,231	No	ns
ACSF infused vs. Chol infused	-5,625	No	ns
Chol contralateral vs. Chol infused	-11,86	No	ns

**FIGURE 6D**			
ANOVA summary			
F	16,24		
P value	<0,0001		
P value summary	****		
Significant diff. among means (P < 0.05)?	Yes		
R squared	0,5076		
Newman-Keuls multiple comparisons test	Mean Diff,	Significant?	Summary
wt vs. ACSF contralateral	-91,86	Yes	****
wt vs. ACSF infused	-129,2	Yes	****
wt vs. Chol contralateral	-91,86	Yes	****
wt vs. Chol infused	-23,15	No	ns
ACSF contralateral vs. ACSF infused	-37,32	No	ns
ACSF contralateral vs. Chol contralateral	0,000008	No	ns
ACSF contralateral vs. Chol infused	68,72	Yes	***
ACSF infused vs. Chol contralateral	37,32	No	ns
ACSF infused vs. Chol infused	106	Yes	****
Chol contralateral vs. Chol infused	68,72	Yes	***

Appendix Figures Legends

Appendix Figure S1 Electron microscopy analysis of myelin sheaths in wt and R6/2 groups. **(A)** Representative EM images of myelinated fibers taken from the striatum of wt, R6/2, R6/2 ACSF and R6/2 chol-high (Scale bar: 500 nm). (**B)** Scatterplot graph of G-ratios against axonal diameters with linear regression. The slope of the best-fit line was significantly higher for R6/2 compared with wt mice, indicating thinner myelin sheaths in HD mice with and without treatment with cholesterol. (**C)** Mean G-ratios were higher in R6/2 mice compared with their wt controls, indicating that HD mice had thinner myelin sheaths (***p< 0.001). At least 300 axons in 3 mice group were subjected to G-ratio analysis. Statistics: one-way ANOVA with Newman–Keuls post-hoc test (*p<0.05; **p<0.01; ***p<0.001). (**D)** Representative EM images of myelinated fibers taken from the corpus callosum of wt, R6/2, R6/2 ACSF and R6/2 chol-high (Scale bar: 500 nm). (**E)** Scatterplot graph of G-ratios against axonal diameters with linear regression. The slope of the best-fit line was significantly higher for R6/2 compared with wt mice, indicating thinner myelin sheaths in HD mice with and without treatment with cholesterol. (**F)** Mean G-ratios were higher in R6/2 mice compared with their wt controls, indicating that HD mice had thinner myelin sheaths (***P < 0.001). At least 300 axons in 3 mice group were subjected to G-ratio analysis. Error bars represent the standard error of the mean. Statistics: one-way ANOVA with Newman–Keuls post-hoc test (*p<0.05; **p<0.01; ***p<0.001). (**G)** Representative EM images of myelinated fibers and its magnification taken from the striatum of wt mice in which the major dense lines in the myelin sheath are indicated. Periodicity was measured as the mean distance between two major dense lines from at least 45 randomly chosen myelin sheaths in 3 mice group. (Scale bars: 30 nm and 10 nm). (**H-I)** Quantification of the mean periodicity in wt, R6/2, R6/2 ACSF and R6/2 chol-high mice. Error bars represent the standard error of the mean. Statistics: one-way ANOVA with Newman– Keuls post-hoc test (*p<0.05; **p<0.01; ***p<0.001).

Appendix Figure S2 MuHTT aggregates in the striatum, cortex and hippocampus of R6/2 chol-high mice.

(A–F) Zoom of representative confocal images of immunostaining against EM48 antibody, specific for muHTT aggregates, in the infused and the contralateral striatum (A), cortex (C) and hippocampus (E) of R6/2 chol-high mice (N=3) and relative quantification (B, D, F) of number of aggregates. Hoechst (Ho, blue) was used to counterstain nuclei. Scale bars in A, C, E: 10 µm. 18 images/animal were analyzed from 9 sections throughout the entire striatum; 10 images for each animal were analyzed from 3 sections of the cortex; 6 images for each animal/condition were analyzed from 3 sections of the hippocampus. All values are expressed as % above the mean of aggregates in the contralateral tissue of R6/2 chol-high mice. The data are shown as scatterplot graphs with means±standard. Each dot corresponding to number of aggregates counted in all the images. Statistics: one-way ANOVA with Newman–Keuls post- hoc test (*p<0.05; **p<0.01; ***p<0.001).
