# Supplementary material for: Striatal infusion of cholesterol promotes dose‐dependent behavioral benefits and exerts disease‐modifying effects in Huntington's disease mice
Source: EMBO Mol Med. 2020 Sep 22;12(10):e12519. doi: 10.15252/emmm.202012519 (PMC7539329; doi:10.15252/emmm.202012519)
Supplement: Supplementary file 2 — Expanded View Figures PDF [file EMMM-12-e12519-s002.pdf]

Expanded View Figures

Figure EV1. Behavioral characterization of wt mice following striatal infusion of the high dose of cholesterol and *in vivo* exogenous cholesterol localization.

A Latency to fall (seconds) from an accelerating rotarod at 10 weeks of age (3 weeks after cholesterol infusion) in wt (N = 11); wt ACSF (N = 11) and wt chol-high (N = 7) mice.

B–E Global motor activity (B), total distance travelled (C), mean velocity (D), and stereotyped movements (E) in an open field at 11 weeks of age (4 weeks after cholesterol infusion) (wt = 11; wt ACSF = 10; wt chol-high = 7).

F Discrimination index (%) in the novel object recognition test of wt, wt ACSF, and wt chol-high mice at 11 weeks of age (4 weeks after cholesterol infusion) (wt = 11; wt ACSF = 10; wt chol-high = 7). DI above zero indicates a preference for the novel object; DI below zero indicates a preference for the familiar object.

G, H Cholesterol content in the infused striatum (G) and ipsilateral cortex (H) of wt ACSF (N = 3), wt chol-low (N = 5), and wt chol-high (N = 4) mice at 12 weeks of age after 4-week striatal cholesterol infusion.

I–M Representative confocal images showing co-localization of BODIPY-chol (green) and TGN46 (I), calnexin (J), Rab9 (K), PMCA-ATPase (L), and LAMP1 (M) (red) in the striatum of R6/2 mice infused with BODIPY-cholesterol. Scale bars: 5 μm.

Data information: The data in (A-H) are shown as scatterplot graphs with means ± standard error. Each dot corresponds to the value obtained from each animal. Values in (A–D) were normalized as % above the mean of wt ACSF for each independent analysis. Statistics: one-way ANOVA with Newman–Keuls *post hoc* test (***$P < 0.001$; ****$P < 0.0001$).

© 2020 The Authors

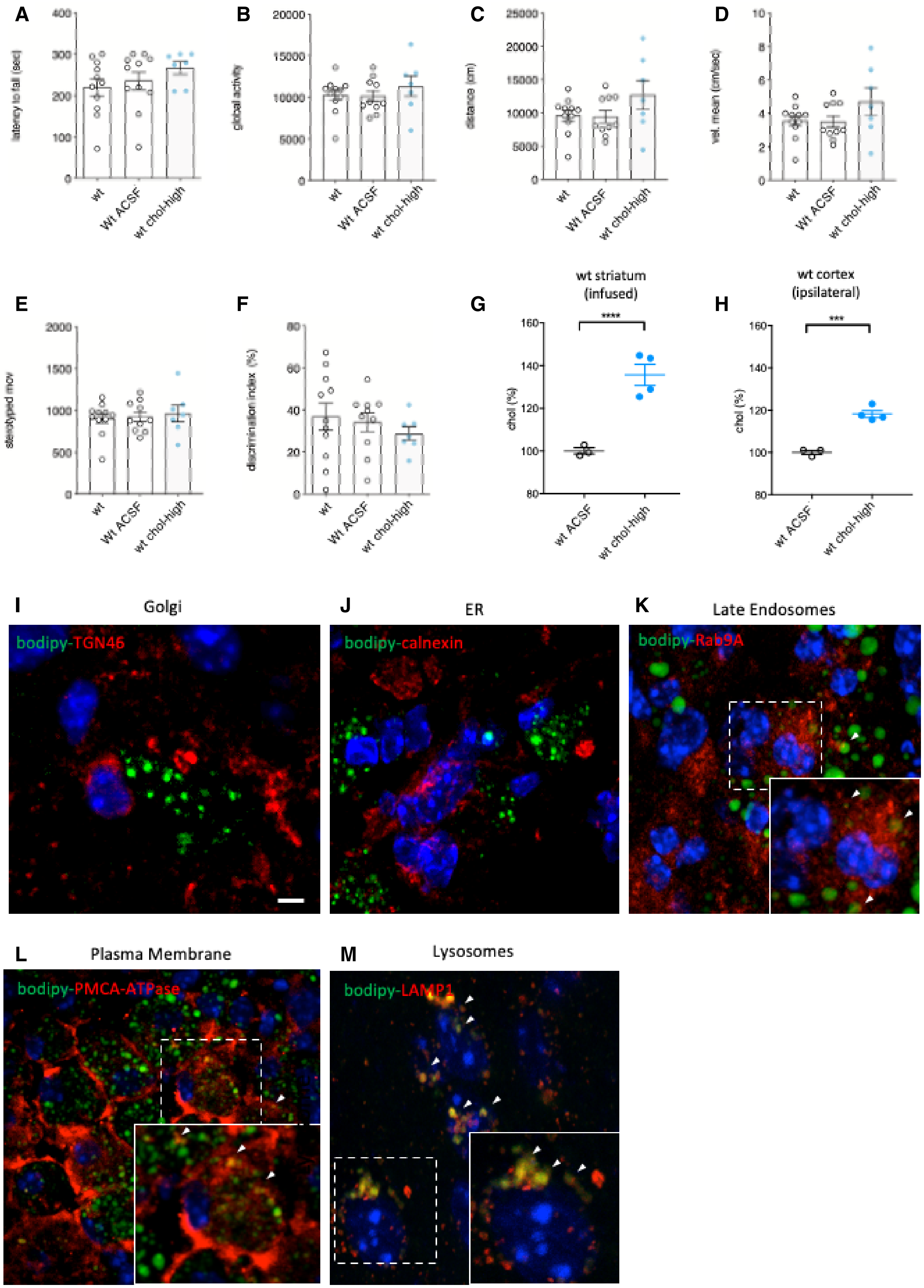
A
B
C
D
E
F
G
H
wt striatum (infused)
wt cortex (ipsilateral)
wt
wt ACSF
wt chol-high
****
***
chol (%)
I
Golgi
bodipy-TGN46
J
ER
bodipy-calnexin
K
Late Endosomes
bodipy-Rab9A
L
Plasma Membrane
bodipy-PMCA-ATPase
M
Lysosomes
bodipy-LAMP1

Figure EV1.

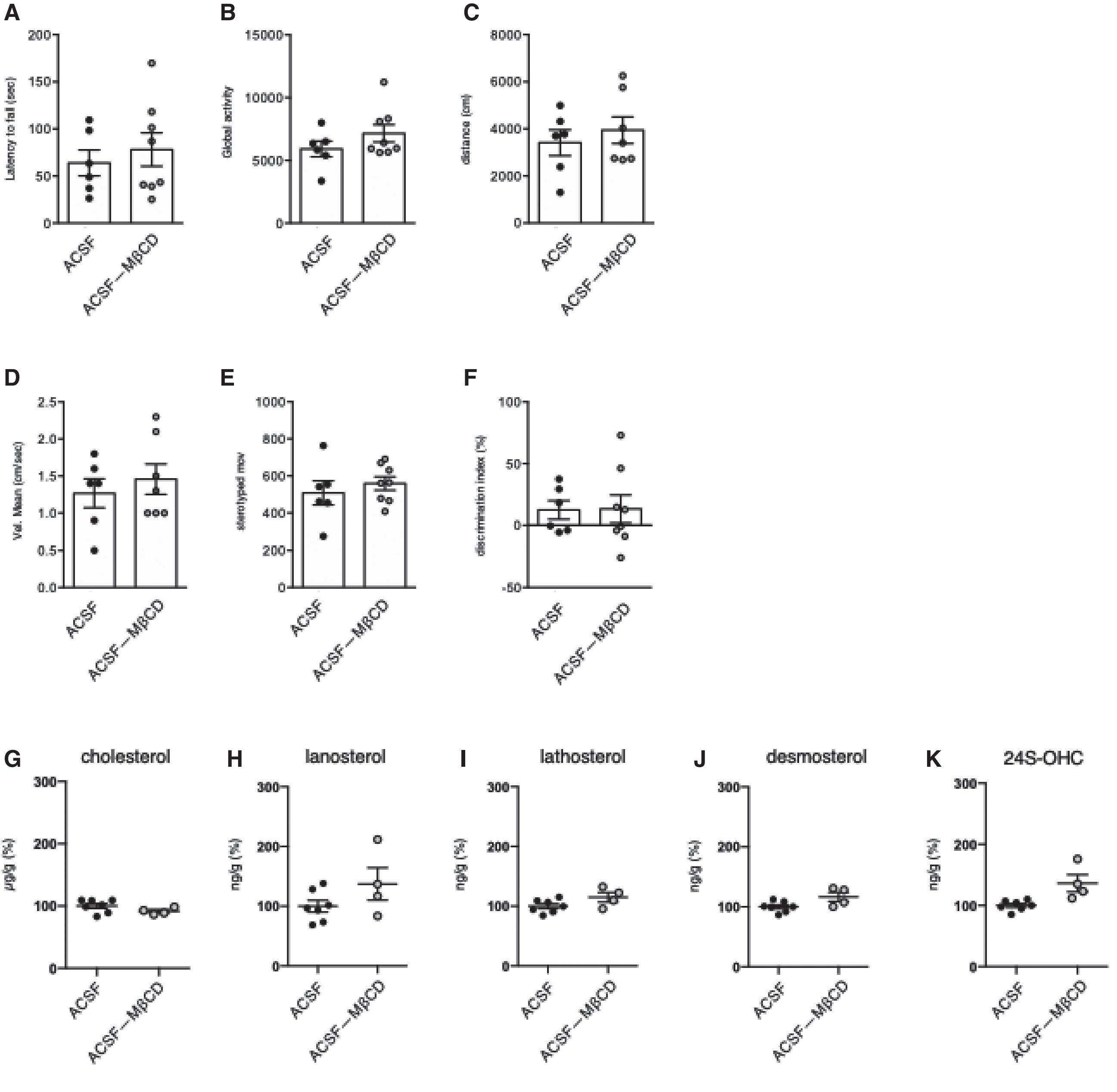

Figure EV2. Behavioral characterization and sterol quantification of R6/2 ACSF and R6/2 ACSF complexed with methyl-β-cyclodextrin.

A Latency to fall (seconds) from an accelerating rotarod at 10 weeks of age (3 weeks after cholesterol infusion) in R6/2 ACSF (*N* = 6) and R6/2 ACSF-MβCD (*N* = 8) mice.

B–E Global motor activity (B), total distance travelled (C), mean velocity (D), and stereotyped movements (E) in an open field at 11 weeks of age (4 weeks after cholesterol infusion) (R6/2 ACSF = 6; R6/2 ACSF-MβCD = 6).

F Discrimination index (%) in the novel object recognition test of R6/2 ACSF and R6/2 ACSF-MβCD mice at 11 weeks of age (4 weeks after cholesterol infusion) (R6/2-ACSF = 6; R6/2 ACSF-MβCD = 8). DI above zero indicates a preference for the novel object; DI below zero indicates a preference for the familiar object.

G–K Cholesterol (G), lanosterol (H), lathosterol (I), desmosterol (J), and 24S-OHC (K) level measured by mass spectrometry in the infused striatum of R6/2 ACSF (*N* = 7) and R6/2 ACSF-MβCD (*N* = 4) mice at 12 weeks of age after 4-week striatal infusion.

Data information: The data in (A–F) are shown as scatterplot graphs with means ± standard error. All values in (G–K) are expressed as % above the mean of R6/2 ACSF, and these data are shown as scatterplots with means ± standard error. Each dot corresponds to the value obtained from each animal.

© 2020 The Authors

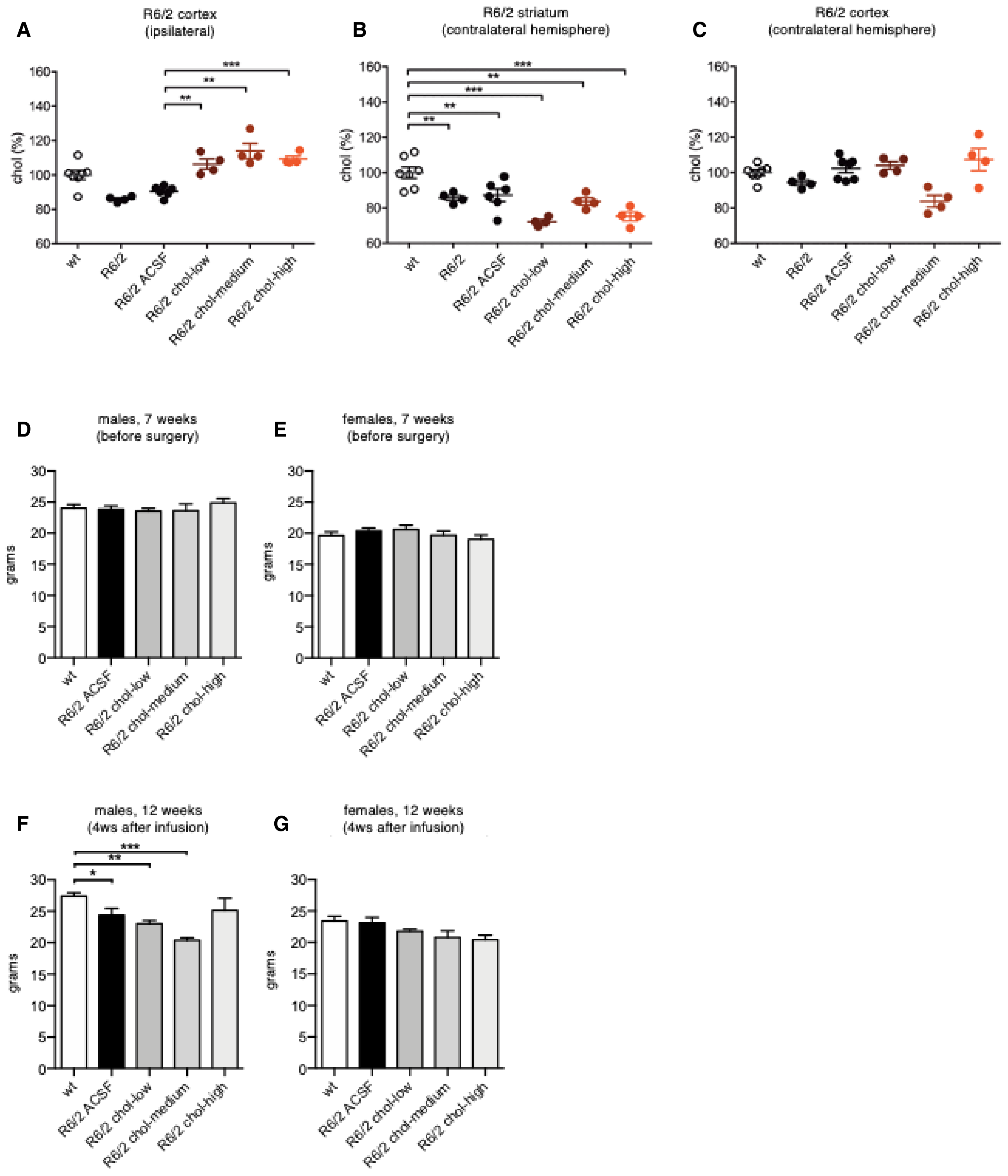

Figure EV3.

© 2020 The Authors

◀

Figure EV3. Cholesterol content in the striatum and cortex of R6/2 mice following striatal infusion of cholesterol and mice body weight.

A–C Cholesterol content in the ipsilateral cortex (A), contralateral striatum (B), and contralateral cortex (C) of wt (N = 7), R6/2 (N = 4), R6/2 ACSF (N = 6), R6/2 chol-low (N = 4), R6/2 chol-medium (N = 4), and R6/2 chol-high (N = 4) mice at 12 weeks of age after 4-week striatal cholesterol infusion.

D–G Body weight (expressed in g) of male (D, F) and female (E, G) mice before surgery (7 weeks of age; wt, N = 19; R6/2 ACSF, N = 24; R6/2 chol-low, N = 18; R6/2 chol-medium, N = 8; R6/2 chol-high, N = 12) and 4 weeks after cholesterol infusion (12 weeks of age; wt, N = 18; R6/2 ACSF, N = 18; R6/2 chol-low, N = 13; R6/2 chol-medium, N = 8; R6/2 chol-high, N = 9).

Data information: The data (A–C) are shown as scatterplot graphs with means ± standard error. Each dot corresponds to the value obtained from each animal. The data (D–G) are shown as histograms with means ± standard error. Statistics: one-way ANOVA with Newman–Keuls *post hoc* test (*P < 0.05; **P < 0.01; ***P < 0.001). All values were normalized as % above the mean of wt for each independent analysis.

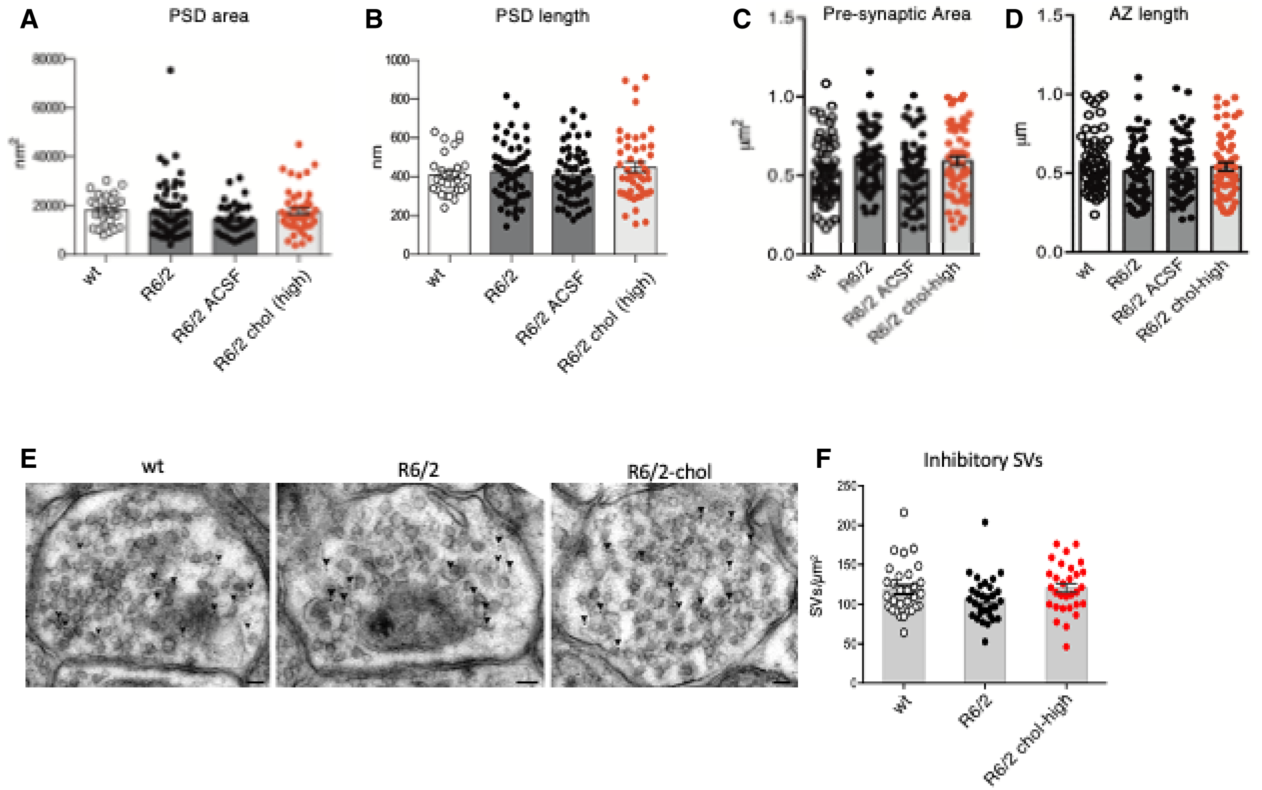

Figure EV4. Morphometric analysis of excitatory synapses and of synaptic vesicle (SV) density of inhibitory synapses following striatal infusion of the high dose of cholesterol.

A–D 60 PSDs (A, B) and 60 pre-synaptic terminals (C, D) were analyzed in 3 mice/group.

E TEM images of inhibitory striatal synapses in wt, R6/2, and R6/2 chol-high mice. GABA-immuno-positive synapses were stained by 12 nm colloidal gold-conjugated secondary antibodies (arrowhead). Scale bar: 50 nm.

F Quantification of SVs density in 30 inhibitory synapses of wt, R6/2 and R6/2 chol-high mice (N = 3 mice/group).

Data information: Graphs are means ± standard error.

© 2020 The Authors

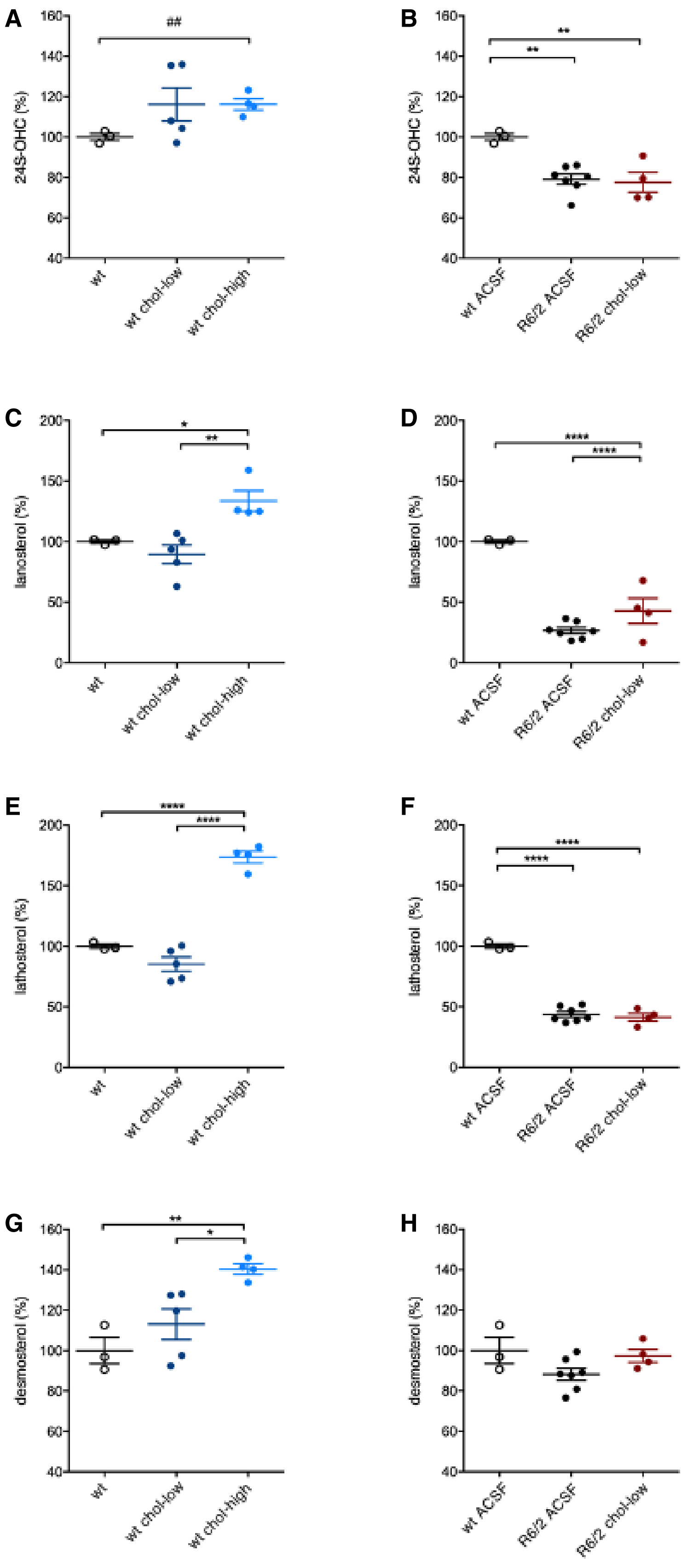

Figure EV5. Levels of cholesterol precursors and 24S-OHC in the striatum of wt and R6/2 groups following striatal infusion of cholesterol.

A, B 24S-OHC level in the infused striatum of wt ACSF ($N = 3$), wt chol-low ($N = 5$), wt chol-high ($N = 4$) (A), and in the infused striatum of wt ACSF ($N = 3$), R6/2 ACSF ($N = 7$), R6/2 chol-low ($N = 4$) (B) at 12 weeks of age after 4-week striatal infusion of cholesterol.

C, D Lanosterol level in the infused striatum of wt ACSF ($N = 3$), wt chol-low ($N = 5$), wt chol-high ($N = 4$) (C), and in the infused striatum of wt ACSF ($N = 3$), R6/2 ACSF ($N = 7$), R6/2 chol-low ($N = 4$) (D) at 12 weeks of age after 4-week striatal infusion of cholesterol.

E, F Lathosterol level in the infused striatum of wt ACSF ($N = 3$), wt chol-low ($N = 5$), wt chol-high ($N = 4$) (E), and in the infused striatum of wt ACSF ($N = 3$), R6/2 ACSF ($N = 7$), R6/2 chol-low ($N = 4$) (F) at 12 weeks of age after 4-week striatal infusion of cholesterol.

G, H Desmosterol level in the infused striatum of wt ACSF ($N = 3$), wt chol-low ($N = 5$), wt chol-high ($N = 4$) (G), and in the infused striatum of wt ACSF ($N = 3$), R6/2 ACSF ($N = 7$), R6/2 chol-low ($N = 4$) (H) at 12 weeks of age after 4-week striatal infusion of cholesterol.

Data information: All values were expressed as % above the mean of wt ACSF. The data of wt ACSF in (A, C, E, G) are the same shown in (B, D, F, H). The data in (A–H) are shown as scatterplot graphs with means ± standard error with each dot corresponding to the value obtained from each animal. Statistics: one-way ANOVA with Newman–Keuls *post hoc* test (*$P < 0.05$; **$P < 0.01$; ***$P < 0.001$), and Student's *t*-test ($^{\#\#}P < 0.01$) in (A).

© 2020 The Authors
