# Supplementary material for: Striatal infusion of cholesterol promotes dose‐dependent behavioral benefits and exerts disease‐modifying effects in Huntington's disease mice
Source: EMBO Mol Med. 2020 Sep 22;12(10):e12519. doi: 10.15252/emmm.202012519 (PMC7539329; doi:10.15252/emmm.202012519)
Supplement: Supplementary file 4 — Table EV2 [file EMMM-12-e12519-s004.docx]

Table EV2. Passive properties of MSNs

|  | **Cm (pF) ± SEM** | **Rin (MΩ) ± SEM** |
| --- | --- | --- |
| **Wt (n=6)** | 68,53±5,72 | 119,96±9,88 |
| **R6/2-ACSF (n=5)** | 51,47±3,75 * | 181,05±20,94 * |
| **R6/2-Chol (n=7)** | 54,13±8,87 | 170,48±15,48 ^§^ |

*wt vs R6/2-ACSF (unpaired two-tailed Student’s t-test; p<0.05)

^§^ wt vs R6/2-Chol (unpaired two-tailed Student’s t-test; p<0.05)
