# Supplementary figures and images for: Striatal infusion of cholesterol promotes dose‐dependent behavioral benefits and exerts disease‐modifying effects in Huntington's disease mice
Source: EMBO Mol Med. 2020 Sep 22;12(10):e12519. doi: 10.15252/emmm.202012519 (PMC7539329; doi:10.15252/emmm.202012519)

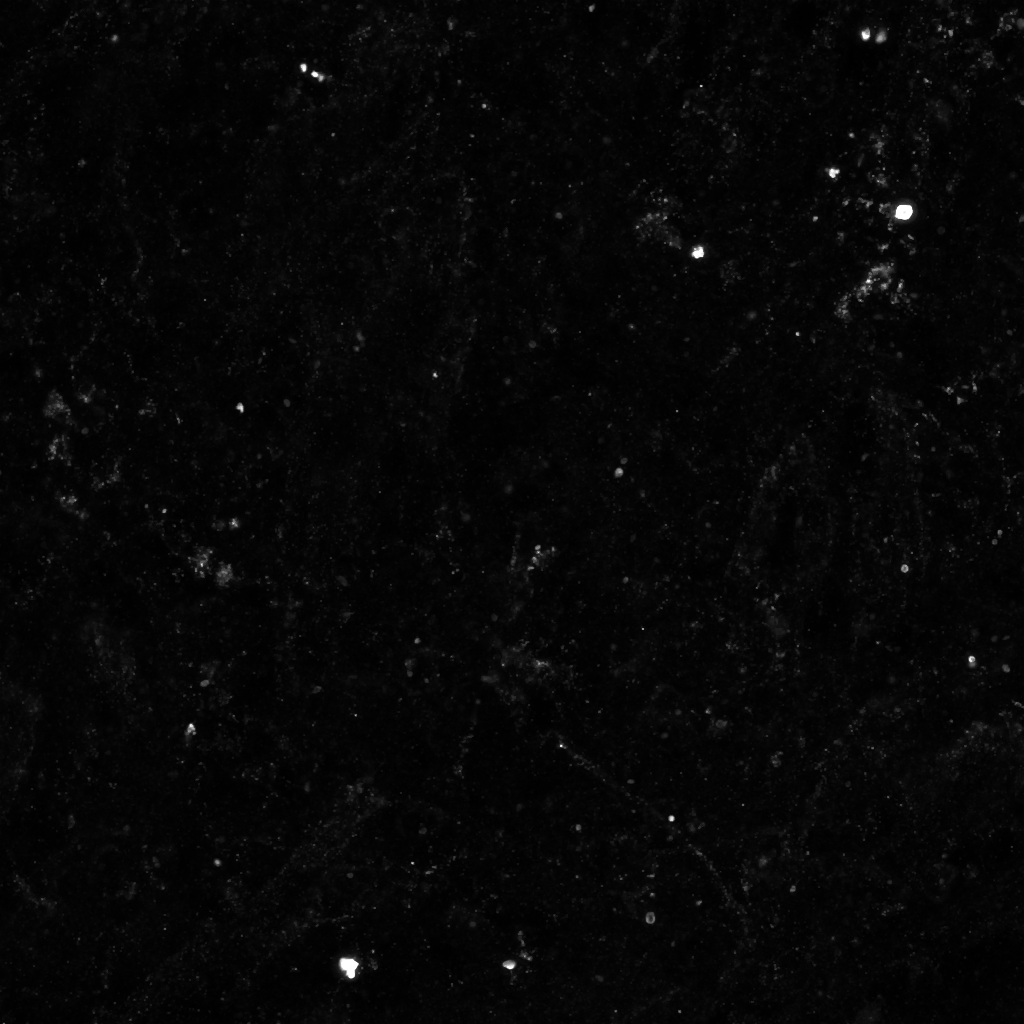

Supplement: Supplementary file 7 — Source Data for Figure 6 [file EMMM-12-e12519-s006.zip › Source data figure 6/R62 acsf contralateral_red.tif]

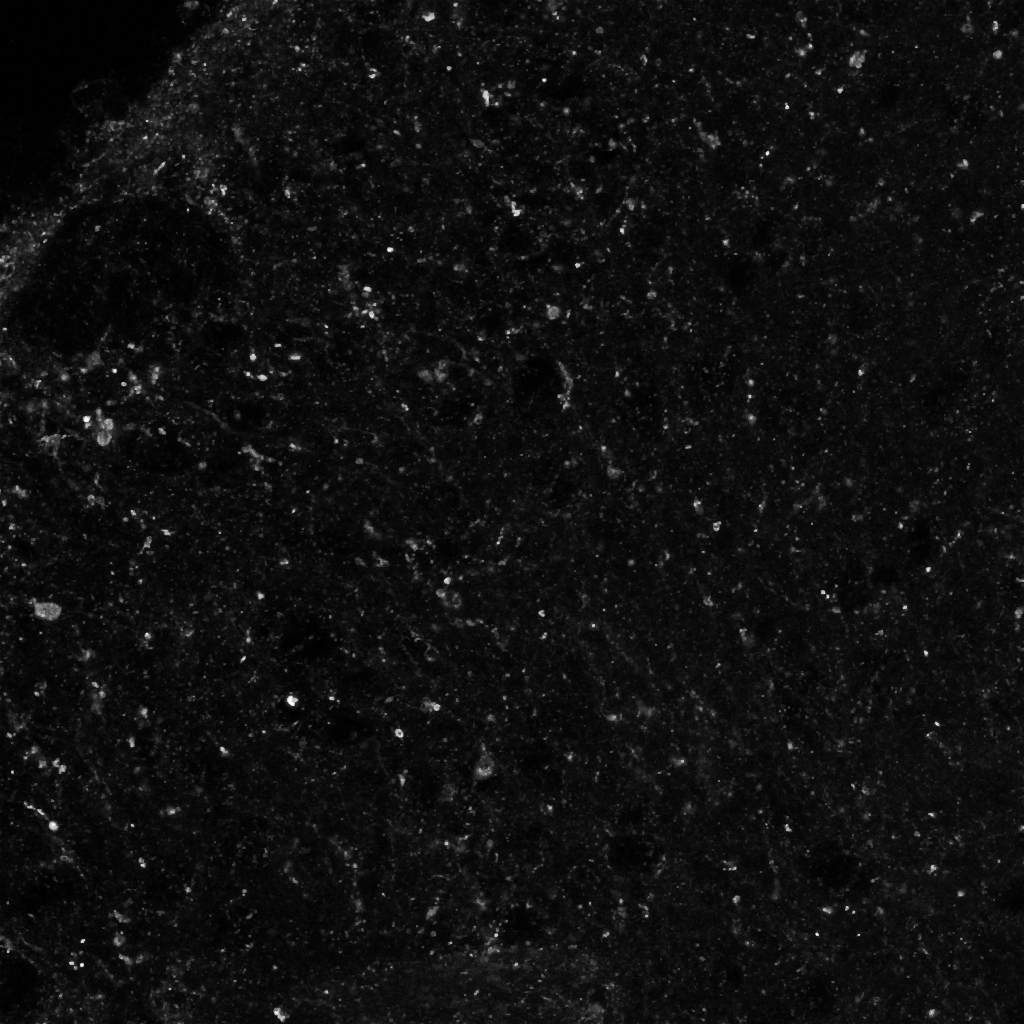

Supplement: Supplementary file 7 — Source Data for Figure 6 [file EMMM-12-e12519-s006.zip › Source data figure 6/R62 chol high contralateral_green.tif]

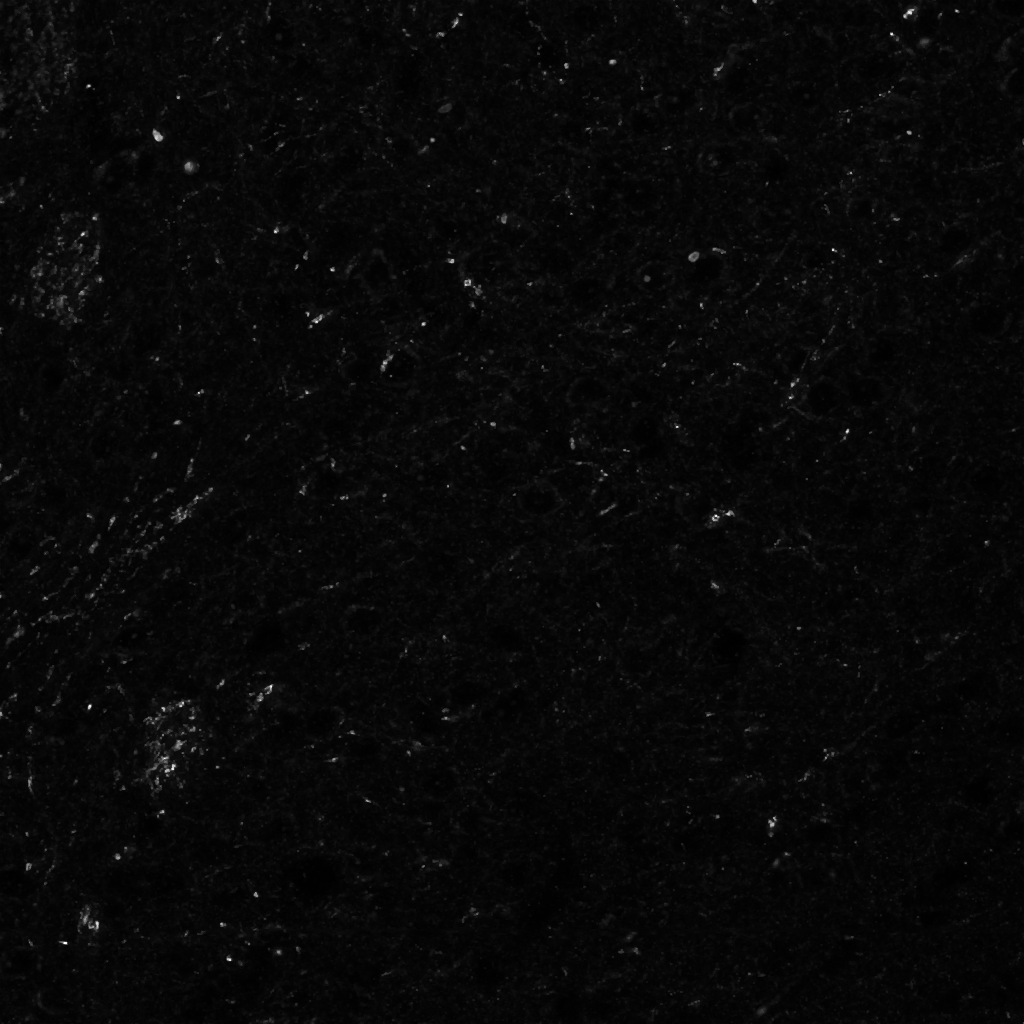

Supplement: Supplementary file 7 — Source Data for Figure 6 [file EMMM-12-e12519-s006.zip › Source data figure 6/R62 chol high infused_red.tif]

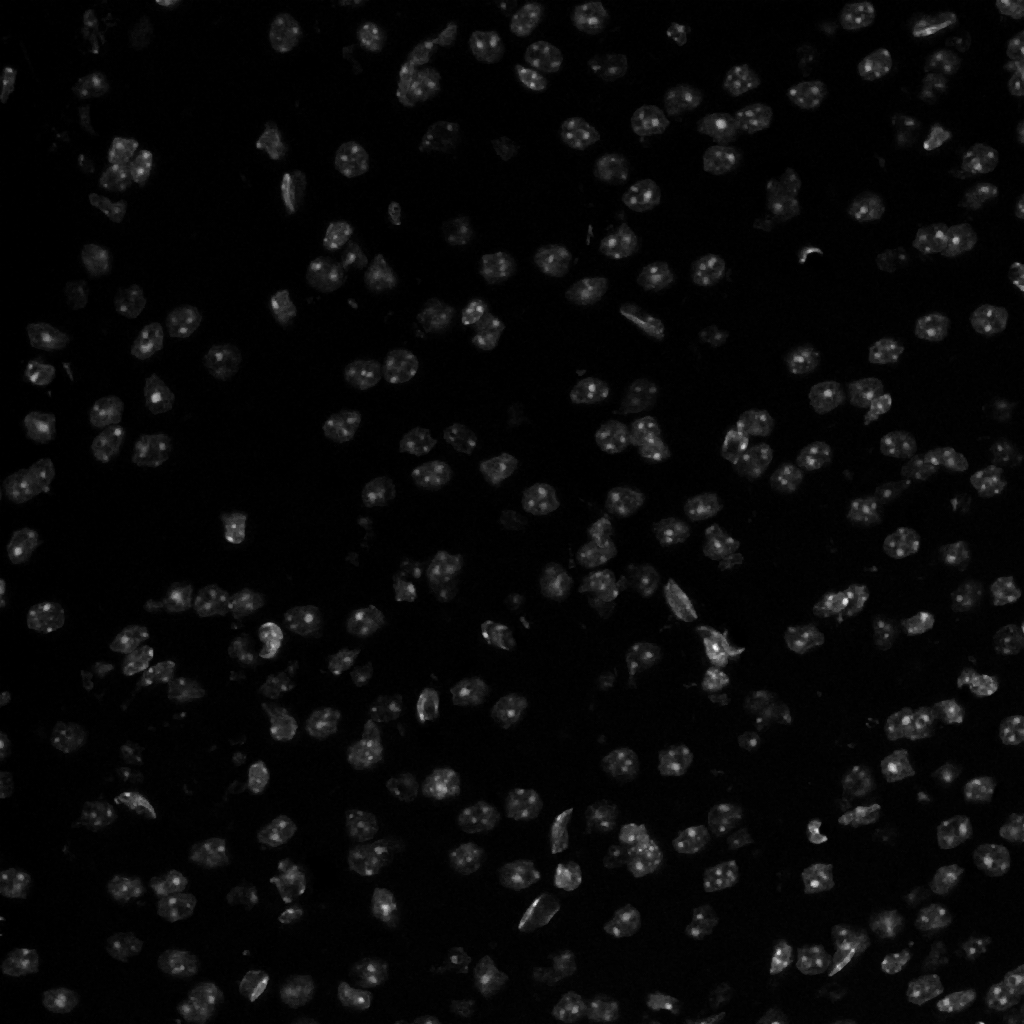

Supplement: Supplementary file 7 — Source Data for Figure 6 [file EMMM-12-e12519-s006.zip › Source data figure 6/R62 chol high infused_blue.tif]

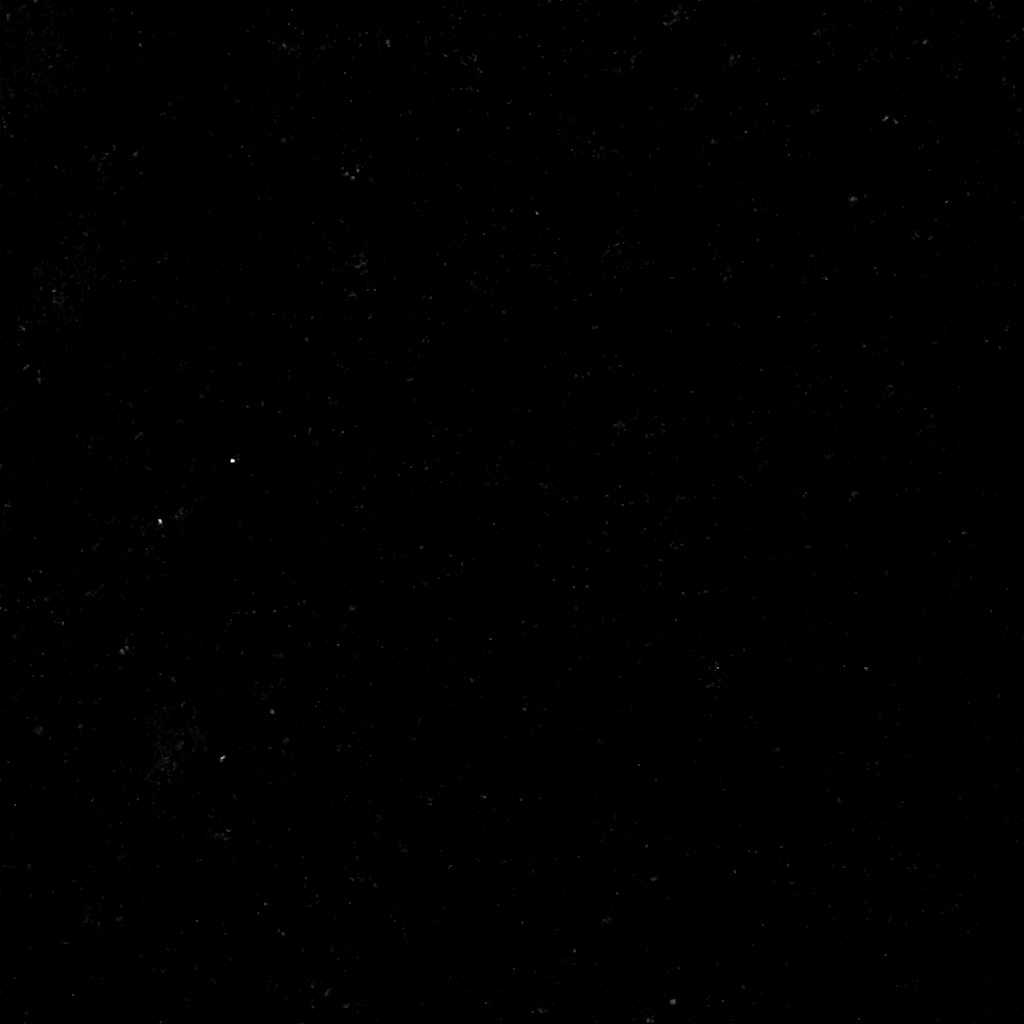

Supplement: Supplementary file 7 — Source Data for Figure 6 [file EMMM-12-e12519-s006.zip › Source data figure 6/R62 chol high infused_green.tif]

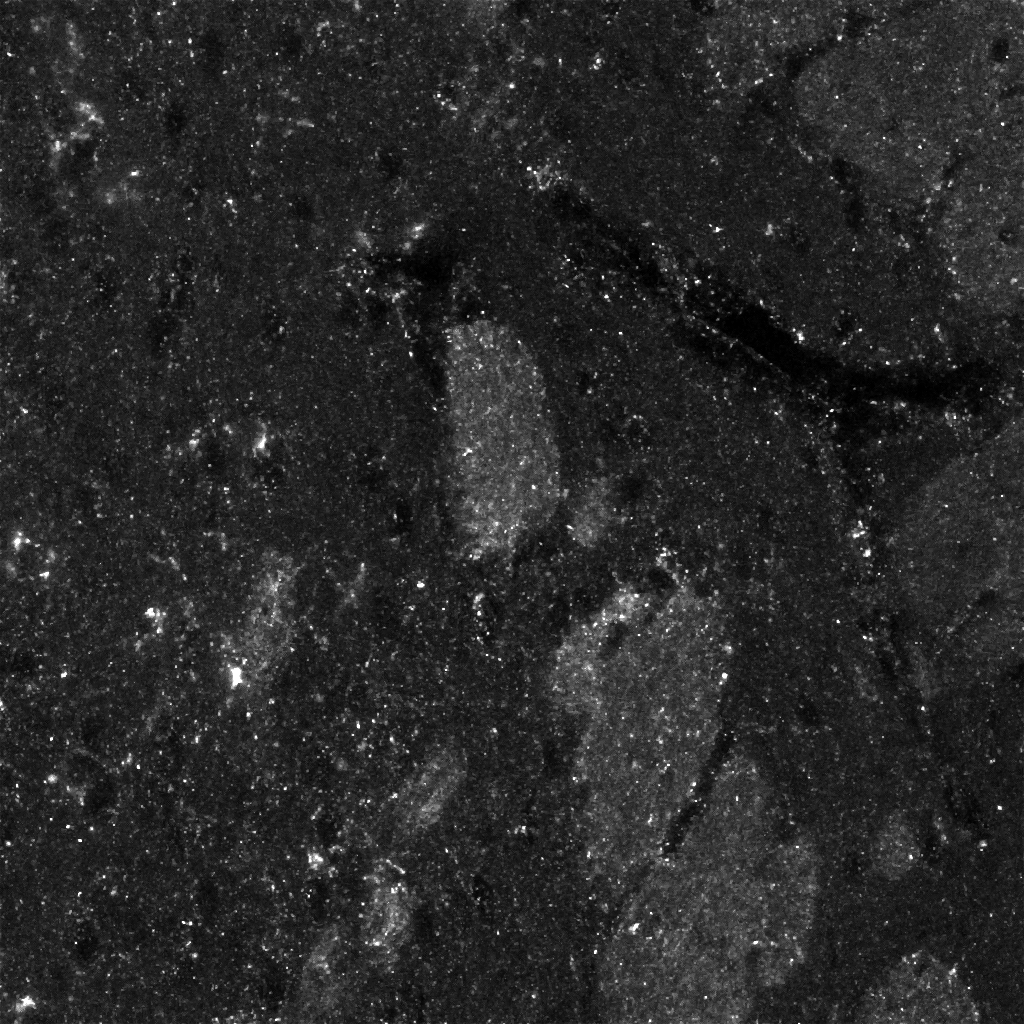

Supplement: Supplementary file 7 — Source Data for Figure 6 [file EMMM-12-e12519-s006.zip › Source data figure 6/R62 acsf infused_green.tif]

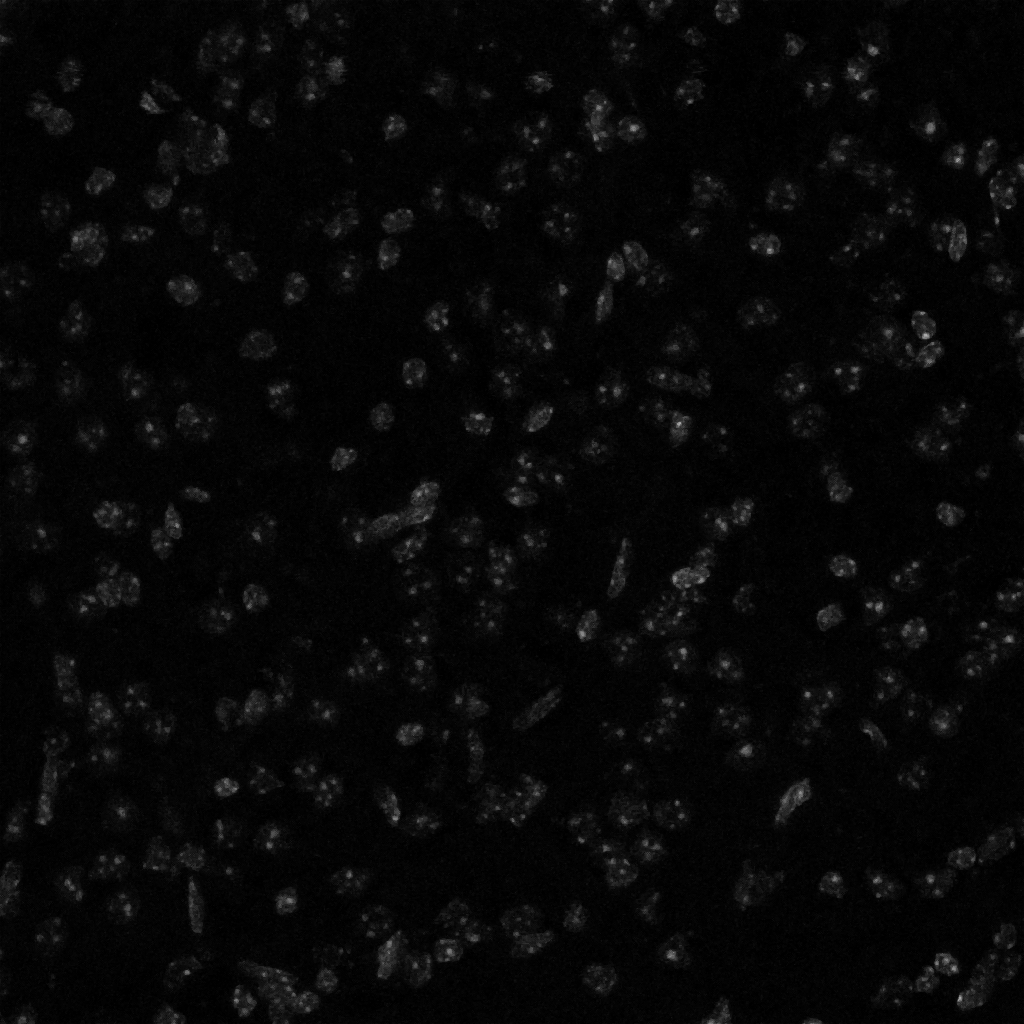

Supplement: Supplementary file 7 — Source Data for Figure 6 [file EMMM-12-e12519-s006.zip › Source data figure 6/R62 acsf contralateral_blue.tif]

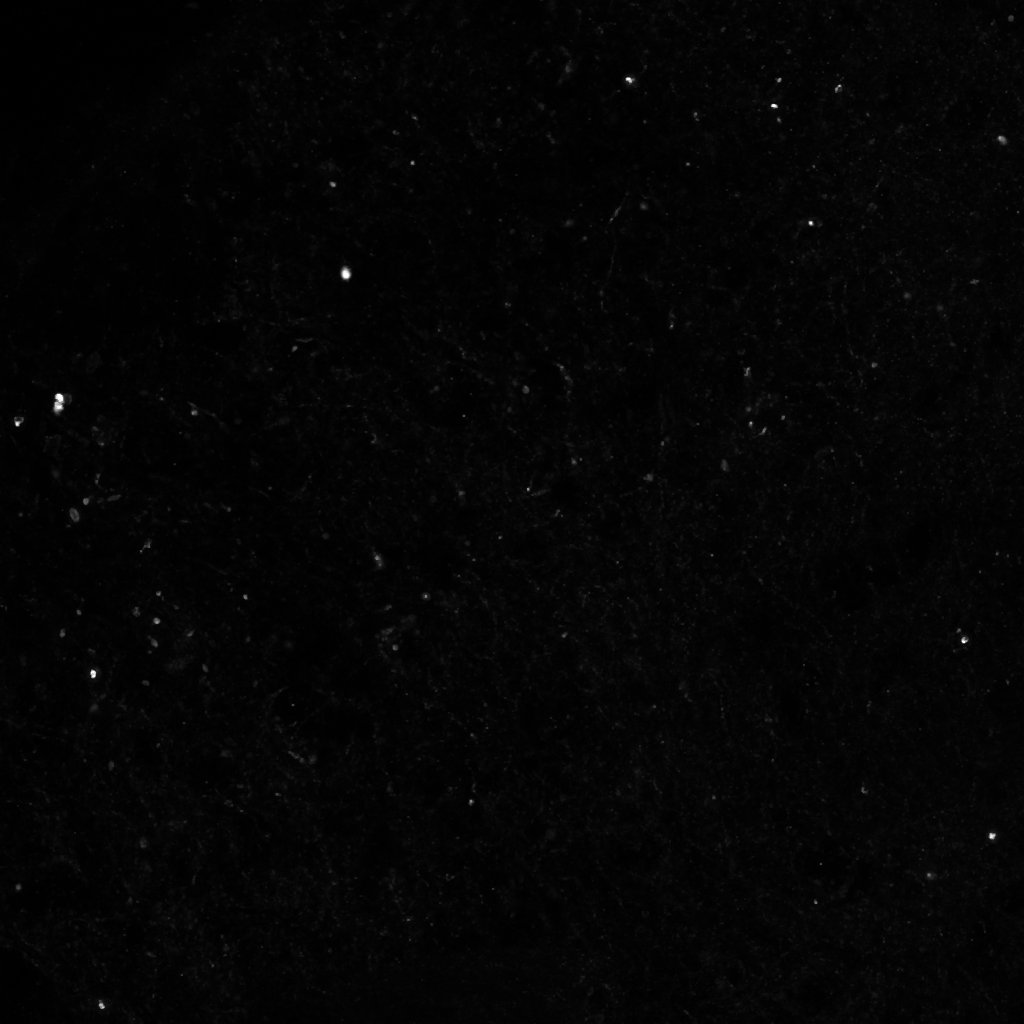

Supplement: Supplementary file 7 — Source Data for Figure 6 [file EMMM-12-e12519-s006.zip › Source data figure 6/R62 chol high contralateral_red.tif]

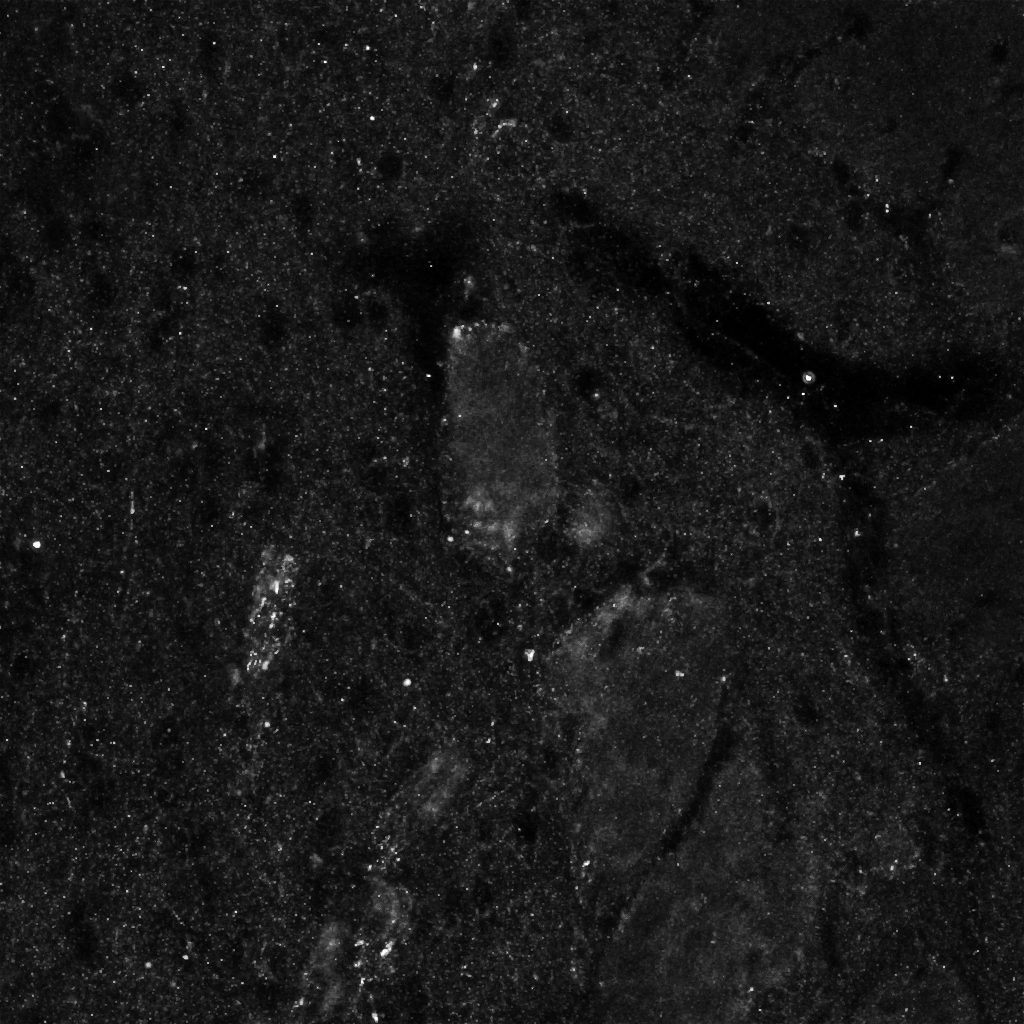

Supplement: Supplementary file 7 — Source Data for Figure 6 [file EMMM-12-e12519-s006.zip › Source data figure 6/R62 acsf infused_red.tif]

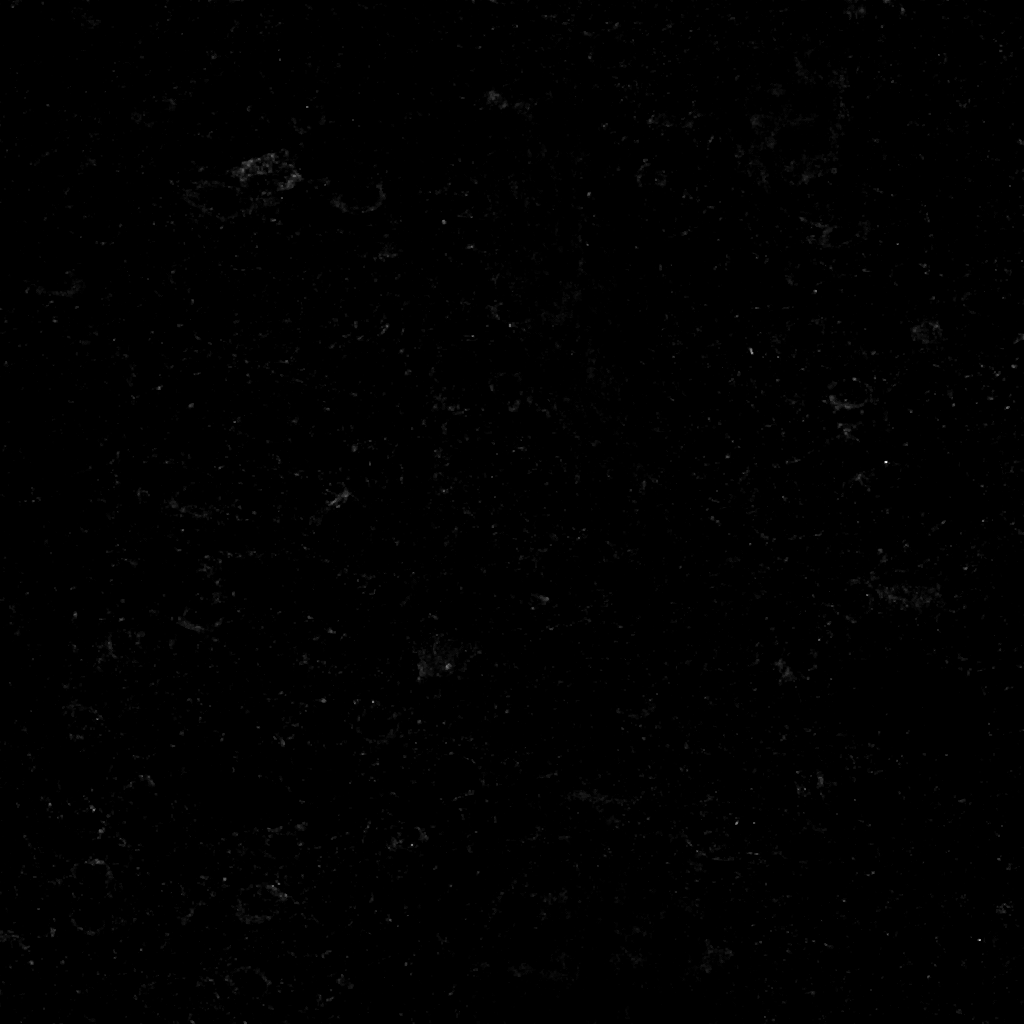

Supplement: Supplementary file 7 — Source Data for Figure 6 [file EMMM-12-e12519-s006.zip › Source data figure 6/wt_red.tif]

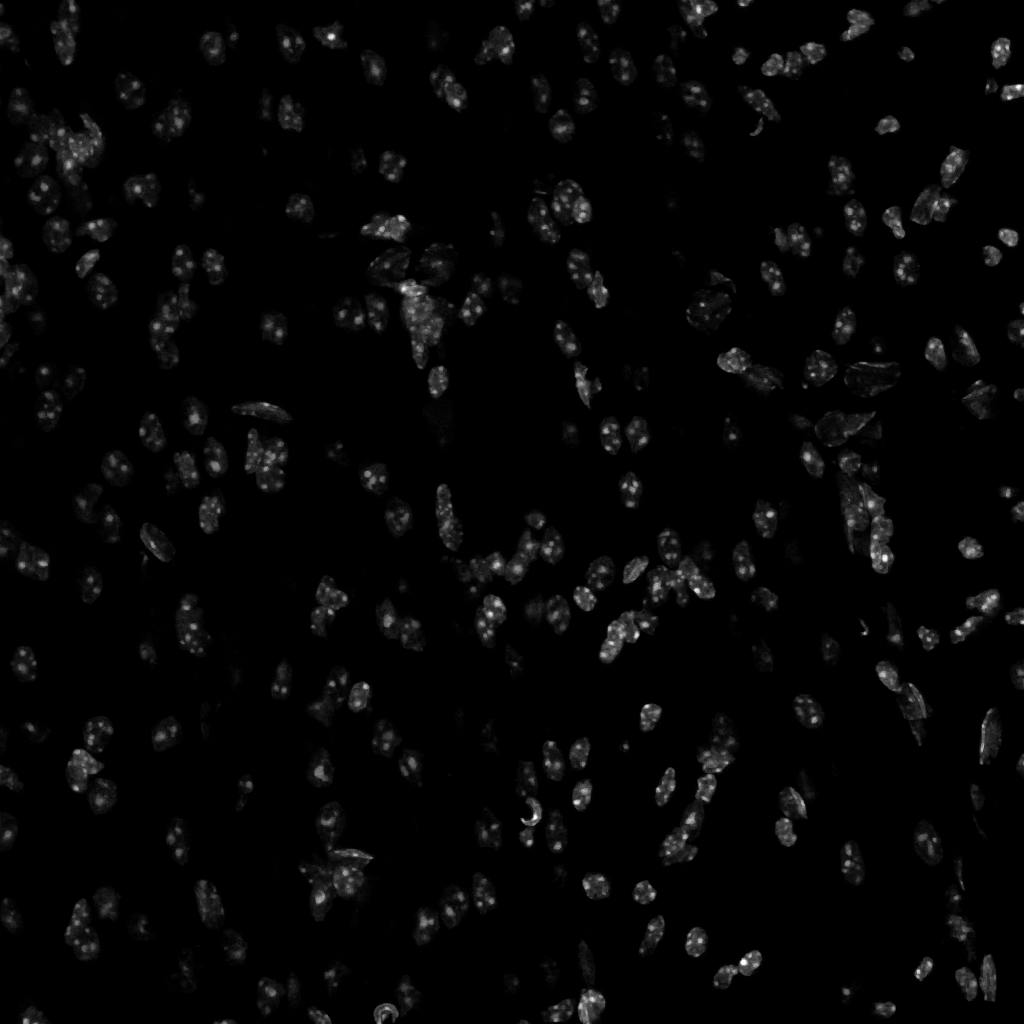

Supplement: Supplementary file 7 — Source Data for Figure 6 [file EMMM-12-e12519-s006.zip › Source data figure 6/R62 acsf infused_blue.tif]

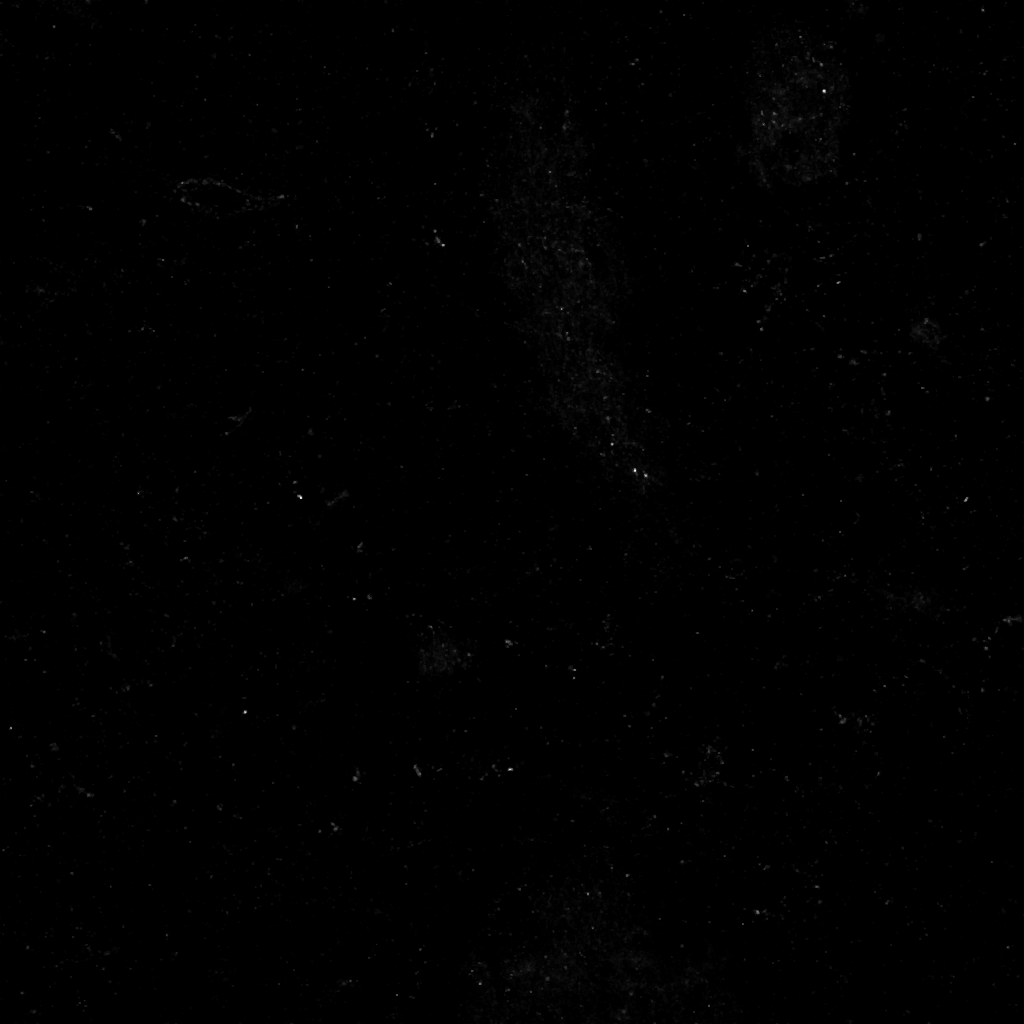

Supplement: Supplementary file 7 — Source Data for Figure 6 [file EMMM-12-e12519-s006.zip › Source data figure 6/wt_green.tif]

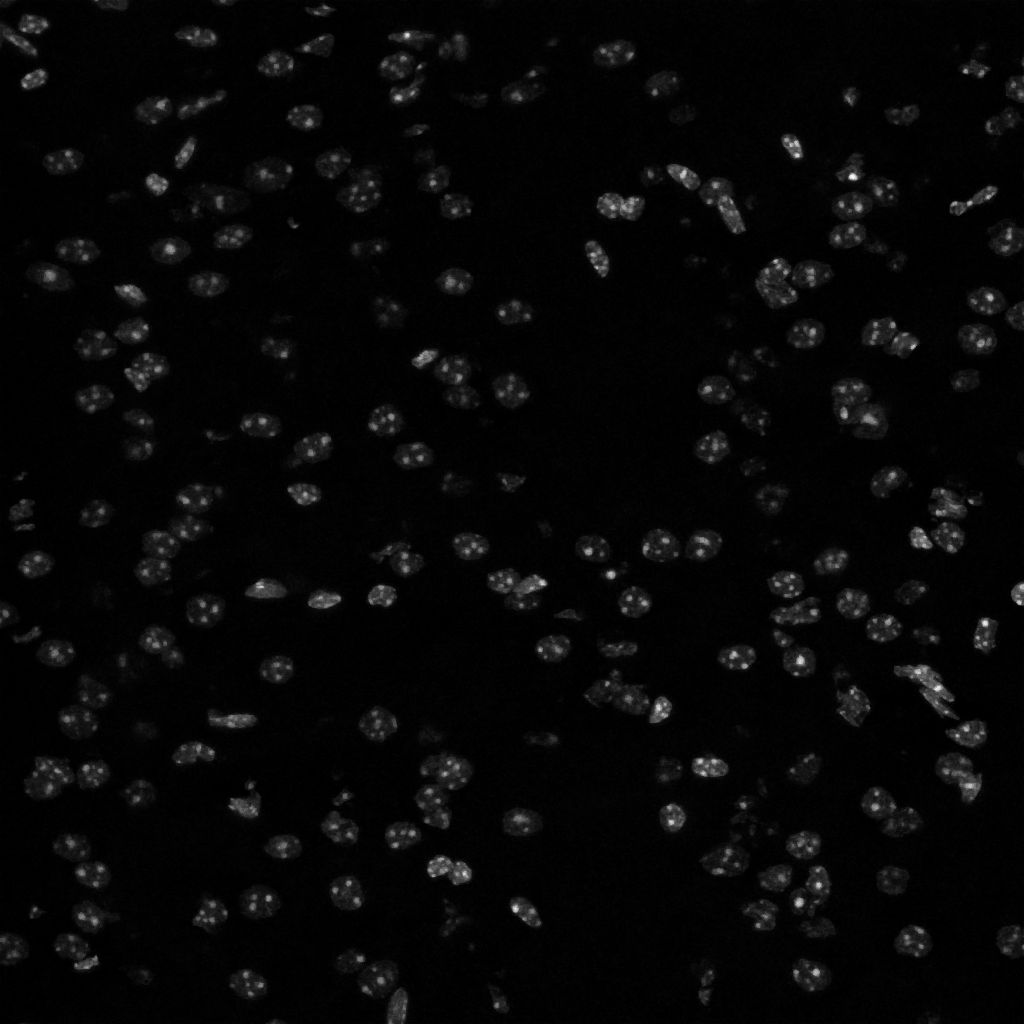

Supplement: Supplementary file 7 — Source Data for Figure 6 [file EMMM-12-e12519-s006.zip › Source data figure 6/wt_blue.tif]

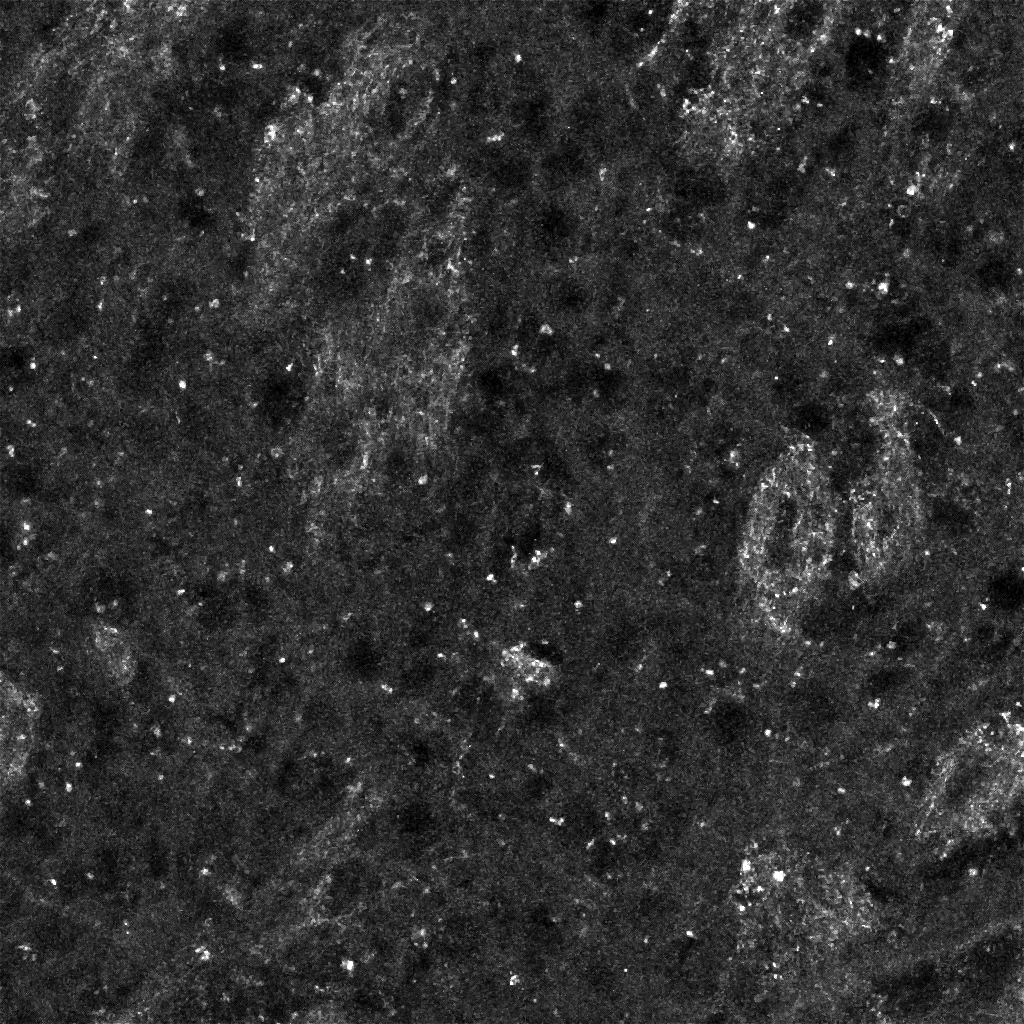

Supplement: Supplementary file 7 — Source Data for Figure 6 [file EMMM-12-e12519-s006.zip › Source data figure 6/R62 acsf contralateral_green.tif]

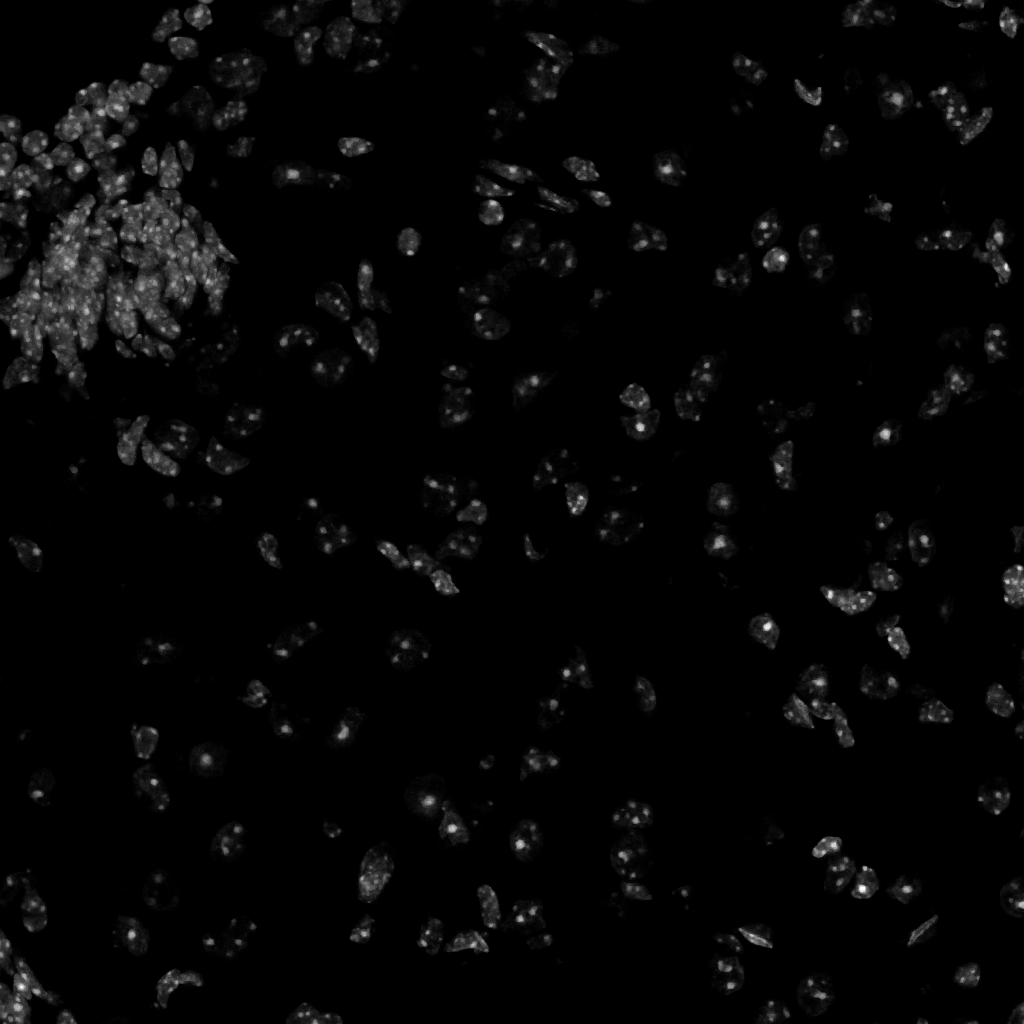

Supplement: Supplementary file 7 — Source Data for Figure 6 [file EMMM-12-e12519-s006.zip › Source data figure 6/R62 chol high contralateral_blue.tif]
